# IRSN-23 gene diagnosis enhances breast cancer subtype classification and predicts response to neoadjuvant chemotherapy: new validation analyses

**DOI:** 10.1007/s12282-025-01687-6

**Published:** 2025-03-24

**Authors:** Yoshiaki Sota, Shigeto Seno, Yasuto Naoi, Keiichiro Honma, Masafumi Shimoda, Tomonori Tanei, Hideo Matsuda, Kenzo Shimazu

**Affiliations:** 1 Department of Breast and Endocrine Surgery, Graduate School of Medicine, Osaka University, 2-15 Yamadaoka, Suita, Osaka 565-0871 Japan; 2 Department of Bioinformatic Engineering, Graduateschool of Information Scienceand Technology, Osaka University, 1-5 Yamadaoka, Suita, Osaka 565-0871 Japan; 3 Department of Surgery, Divisionof Endocrineand BreastSurgery, Kyoto Prefectural University of Medicine, 465 Kawaramachi-hirokoji, Kamigyo-ku, Kyoto, 602-8566 Japan; 4 Department of Diagnostic Pathology and Cytology, Osaka International Cancer Institute, 3-1-69 Otemae, Chuo-ku, Osaka, Osaka 541-8567 Japan; 5 Department of Breast and Endocrine Surgery, Osaka University Graduate School of Medicine, 2-2-E10 Yamadaoka, Suita, Osaka 565-0871 Japan

**Keywords:** Breast cancer, Immune signature, Neoadjuvant chemotherapy, Pathological complete response, Intrinsic subtype

## Abstract

**Background:**

This study evaluates the reproducibility of the IRSN-23 model, which classifies patients into highly chemotherapy-sensitive (Gp-R) or less-sensitive (Gp-NR) groups based on immune-related gene expression using DNA microarray analysis, and its impact on breast cancer subtype classification.

**Methods:**

Tumor tissues from 146 breast cancer patients receiving neoadjuvant chemotherapy (paclitaxel-FEC) ± trastuzumab at Osaka University Hospital (OUH) were used to classify patients into Gp-R or Gp-NR using IRSN-23. The ability to predict a pathological complete response (pCR) was assessed and the results were validated with independent public datasets (N = 1282).

**Results:**

In the OUH dataset, the pCR rate was significantly higher in the Gp-R group than in the Gp-NR group without trastuzumab (29 versus 1%, P = 1.70E−5). In all validation sets without anti-HER2 therapy, the pCR rate in the Gp-R group was significantly higher than that in the Gp-NR group. The pooled analysis of the validation set showed higher pCR rates in the Gp-R group than in the Gp-NR group, both without (N = 1103, 40 versus 12%, P = 2.02E−26) and with (N = 304, 49 versus 35%, P = 0.017) anti-HER2 therapy. Collaboration analyses of IRSN-23 and Oncotype Dx or PAM50 could identify highly chemotherapy-sensitive groups and refine breast cancer subtype classification based on the tumor microenvironment (offensive factor—PAM50 and defensive factor—IRSN-23), and the immune subtype was correlated with a better prognosis after NAC.

**Conclusions:**

This study offers new validation analyses of IRSN-23 in predicting chemotherapy efficacy, showing high reproducibility. The findings indicate the clinical value of using IRSN-23 for refining breast cancer subtype classification, with implications for personalized treatment strategies and improved patient outcomes.

**Supplementary Information:**

The online version contains supplementary material available at 10.1007/s12282-025-01687-6.

## Introduction

Neoadjuvant chemotherapy (NAC) has been widely used in clinical practice for advanced breast cancer since its introduction in a clinical trial more than 40 years ago [1]. The primary objectives of NAC are to enhance breast conservation rates and determine drug sensitivity [2]. Recently, “response-guided therapy” has gained prominence, in which postsurgical drug treatment is adjusted based on the presence or absence of residual tumors [3, 4]. This approach has been increasingly recognized for its significance and has been demonstrated in various trials. The pathological complete response (pCR) has been identified as the factor most strongly associated with prognosis in advanced breast cancer [5], particularly in HER2-positive [6] and triple-negative breast cancer [7]. However, the pCR rate following NAC remains relatively low at 30–50% [2]. Accurate prediction of treatment response before therapy is desirable; however, despite the investigation of several response predictors, a clinically appropriate level of testing has not yet been developed.

Previous studies [8–15] have attempted to predict the efficacy of anthracyclines and taxanes; however, these methods have not been adopted in clinical practice because of the insufficient accuracy and reproducibility of efficacy prediction. Consequently, there is a need for efficient mathematical combinations of sophisticated genetic markers to achieve highly accurate predictions. In 2014, we introduced the IRSN-23 model (**I**mmune-**R**elated Gene **S**ignature for **N**AC, **23** probes) [16], which classifies patients receiving anthracycline and/or taxane without anti-HER2 therapy into two groups based on the expression patterns of 23 probes (19 immune-related genes) using the Affymetrix Human Genome U133 Plus 2.0 chip, which is highly sensitive to chemotherapy (Gp-R, immune score (IS) > 0) and less sensitive to chemotherapy (Gp-NR, IS ≤ 0). Through retrospective validation using in-house data and a large number (n = 901) of public datasets, we demonstrated that the model can predict chemotherapy sensitivity with high accuracy and statistical significance for all breast cancer subtypes.

This study aimed to analyze newly collected data from Osaka University Hospital (OUH, n = 146) and over 1,282 freshly uploaded public data from the past decade after developing the IRSN-23 gene signature as an independent validation set. The primary objective was to demonstrate the reproducibility and clinical utility of the IRSN-23 model. The secondary objective was to evaluate the correlation between IRSN-23 and clinicopathological factors, including various immunohistochemical (IHC) staining and whole-exome sequencing (WES) results, and its impact on breast cancer subtypes. Our results indicated that IRSN-23 has high predictive accuracy for chemotherapy sensitivity and can reliably identify patients who are likely to benefit from NAC without anti-HER2 therapy (previous report (n = 901): Gp-R: 39% vs. Gp-NR: 11%, P = 4.98E−23; current report (n = 1103): Gp-R: 40% vs. Gp-NR: 12%, P = 1.24E−26). Our study underscores the potential clinical application of IRSN-23 in identifying new breast cancer subtypes that are responsive to treatment and could contribute to the personalized treatment of patients with breast cancer.

## Methods

### Patients and samples

#### OUH dataset

Tumor specimens were collected from patients with breast cancer (stages II-IV, n = 146) who received NAC at the Osaka University Hospital (OUH) between 2010 and 2017. Gene expression analysis was performed using Affymetrix U133A Plus 2.0 DNA microarray since the last publication. The chemotherapy regimen involved 12 weekly doses of paclitaxel (80 mg/m^2^), followed by four cycles of 5-FU (500 mg/m^2^), epirubicin (75 mg/m^2^), and cyclophosphamide (500 mg/m^2^) every three weeks. The HER2-positive patients were treated with paclitaxel (PTX) and trastuzumab. Table 1 provides the details of the patients’ backgrounds. The pathological responses were assessed using surgical specimens obtained after chemotherapy. All patients provided informed consent before tumor biopsy, and the Ethics Committee approved the study (Approval Number: K14190). Approval No.K14190 is an observational study designed to develop a drug-sensitivity diagnostic method based on gene expression and mutation analyses of tumor tissues collected before NAC. This study specifically focused on patients with stage I-IV (In the case of Stage IV, only micro metastasis is allowed to be entered) breast cancer scheduled to receive NAC. RNA extraction and DNA microarray analysis methods have been described previously [16].

**Table 1:** Clinicopathological characteristics

	Osaka university hospital			External validation from GEO										
	(Already reported)		Prospective	(Already reported)	Unreported									
	Training	Validation I	Validation II	Ann.Oncol.2014	GSE25066	GSE28844	GSE37946	GSE42822	GSE66399	GSE140494	GSE4779	GSE21974	GSE130788	GSE34138
No. of patients														
	58	59	146	901	508	32	50	91	88	91	102	32	110	178
Summary of data														
Platform	GPL570	GPL570	GPL570	GPL96,570,571	GPL96	GPL570	GPL570	GPL570	GPL570	GPL570	GPL1352	GPL6480	GPL6480	GPL6884
Age														
> 50	31	29	86	394	231	18	25	38	0	49	0	0	0	0
≤ 50	27	30	59	507	277	14	25	53	0	42	0	0	0	0
Unknown	0	0	0	0	0	0	0	0	88	0	102	32	110	178
Tumor size														
0	0	0	1	6	3	0	0	0	0	0	0	0	0	0
1	2	3	14	52	30	0	0	1	0	5	2	7	0	0
2	46	42	93	507	255	0	0	34	0	65	63	25	0	0
3	9	9	21	222	145	0	0	47	0	15	34	0	0	0
4	1	5	17	113	75	0	0	9	0	6	0	0	0	0
Unknown	0	0	0	0	0	32	50	0	88	0	3	0	110	178
cN														
Negative	17	15	49	217	157	0	0	29	0	47	37	0	0	0
Positive	41	44	97	403	351	0	0	59	0	43	62	0	0	0
Unknown	0	0	0	245	0	32	50	3	88	1	3	32	110	178
ER														
Positive	36	37	101	407	297	0	18	38	0	60	37	18	62	119
Negative	22	22	45	494	205	0	32	52	0	31	65	14	48	57
Unknown	0	0	0	0	6	32	0	1	88	0	0	0	0	2
HER2														
Positive	17	18	49	114	6	0	50	34	88	17	0	8	110	0
Negative	41	41	97	761	485	0	0	54	0	74	0	24	0	178
Unknown	0	0	0	26	17	32	0	3	0	0	102	0	0	0
HG														
1 or 2	45	51	76	252	212	0	0	23	0	45	0	16	0	0
3	13	8	70	348	259	0	0	53	0	44	0	16	0	0
Unknown	0	0	0	301	37	32	50	15	88	2	102	0	110	178
Chemotherapy														
A + T	58	59	146	346	508	32	0	91	88	91	0	0	0	0
A	0	0	0	283	0	0	0	0	0	0	102	32	0	178
T	0	0	0	0	0	0	0	0	0	0	0	0	110	0
Unknown	0	0	0	0	0	0	50	0	0	0	0	0	0	0
Anti-HER2 therapy														
Trastuzumab	0	0	34	0	0	0	50	25	23	0	0	0	31	0
Trastuzumab + Lapatinib	0	0	0	0	0	0	0	0	34	0	0	0	50	0
Lapatinib	0	0	0	0	0	0	0	0	31	0	0	0	29	0
None	58	59	112	901	508	32	0	66	0	91	102	32	0	178
Therapeutic effect														
pCR	18	9	37	198	99	4	27	37	27	23	39	8	47	28
Non-pCR	40	50	109	703	389	28	23	54	61	68	63	24	63	149
Unknown	0	0	0	0	20	0	0	0	0	0	0	0	0	1
Sequence analyses														
Whole exome	15	17	81	–	–	–	–	–	–	–	–	–	–	–
Whole RNA	–	–	43	–	–	–	–	–	–	–	–	–	–	–

*Ann.Oncol.2014* external validation set reported in annals of oncology in 2014, *cN* clinical nodal status, *ER* estrogen receptor, *HER2* human epidermal growth factor receptor 2, *HG* histological grade, *wP-FEC* weekly paclitaxel (WP) followed by FEC, *AC* adriamycin plus cyclophosphamide, *EC* epirubicin plus cyclophosphamide, *TC* docetaxel plus cyclophosphamide, *UFT* + *LV* tegafur-uracil plus leucovorin calcium, *pCR (ypT0/N0)* absence of invasive cancer and in-situ cancer in the breast and axillary nodes, *GEO* gene expression omnibus, *GSE* series accession number in GEO, *GPL* platform accession number in GEO, *GPL570* Affymetrix Human Genome U133 Plus 2.0 Array, *GPL571* Affymetrix Human Genome U133A 2.0 Array, *GPL96* Affymetrix Human Genome U133A Array, *GPL6884* Illumina HumanWG-6 v3.0 expression beadchip, *GPL1352* Affymetrix Human X3P, *GPL6480* Agilent-014850 Whole Human Genome Microarray 4 × 44 K G4112F

#### Public dataset

To ensure the validity of IRSN-23 predictions, we re-evaluated their accuracy using available datasets from the Gene Expression Omnibus (GEO) downloaded through January 2022, following the methodology described in our previous publication [16]. We applied the following eligibility criteria: (i) total RNA expression data were obtained from DNA microarrays before treatment; (ii) the dataset had objective efficacy determination following treatment, and the results were retrieved; (iii) the dataset did not report IRSN-23 results; and (iv) the dataset did not include RNA extracted from formalin-fixed paraffin-embedded (FFPE) specimens. Previous studies have demonstrated that RNA extraction from FFPE tissues significantly alters immune-related gene expression. In contrast to our previous analysis [16], this study preoperatively included patients who received neoadjuvant anti-HER2 therapy for breast cancer. The external validation set consists of 1282 cases from eight datasets (GSE25066, GSE28844, GSE66399, GSE140494, GSE21974, GSE130788, GSE4779, and GSE34138). Twenty-one cases were excluded due to unknown efficacy following chemotherapy, including 1261 cases for analysis. The patient characteristics are summarized in Table 1.

### Experiment design

#### Experiment 1: validation of reproducibility

This study aimed to validate the reproducibility of IRSN-23, a gene expression signature composed of 23 probes targeting 19 genes, to predict treatment efficacy in patients with cancer. To accomplish this, we evaluated the performance of IRSN-23 using microarray data from 146 patients, including 34 who received trastuzumab from our institution and 860 patients from GSE25066, GSE28844, GSE37946, GSE42822, GSE66399, and GSE140494, all of which utilized the Human Genome U133 Plus 2.0 platform, for external validation.

To assess the robustness of IRSN-23, we collected microarray data from 320 patients, including 110 from various platforms, such as GSE4779 using the Affymetrix Human X3P Array, GSE21974, and GSE34138 using RNA.Seq-014850 Whole Human Genome Microarray 4 × 44 K G4112F, and GSE130788 using the Illumina Human WG-6 v3.0 expression bead chip and compared their accuracy.

Furthermore, IRSN-23 calculated via microarray was measured using RNA-Seq (n = 43), and their concordance rates were compared.

#### Experiment 2: predictors of chemotherapy sensitivity and clinical utility

IRSN-23 and each factor (clinical factors and factors by IHC, TMB, and HRD by WES) were compared regarding sensitivity to chemotherapy. In addition, IRSN-23 IS collaborated with Oncotype Dx RS for ER + HER2− subtype to investigate its clinical usefulness.

#### Experiment 3: characterization of IRSN-23 and its impact on breast cancer subtype classification

In addition to IRSN-23 and clinicopathological factors, this study also investigated the presence or absence of various immune cells, including tumor-infiltrating lymphocytes (TILs), regulatory T cells (Foxp3-positive T cells), CD8 + T cells, and IL17F + cells (helper T cells), as well as the pre-treatment neutrophil–lymphocyte ratio (NLR), pre-treatment platelet-to-lymphocyte ratio (PLR), HRD score obtained from WES analysis, and TMB. The number of cases included in the study has increased since the previous report, with 117 training and validation cases reported in a previous report. Furthermore, we examined the effects of IRSN-23 on breast cancer subtype classification.

### Measurement methods for each factor

#### Immune-related 23 gene signature for NAC using DNA microarray

In a previous study [16], we developed the IRSN-23, which consists of 23 probes (19 genes) and is now available on GitHub (https://github.com/SNlaboratory/irsn23), and using supervised analysis with DLDA (Diagonal Linear Discriminant Analysis) in the R package ‘supclust’ (https://github.com/cran/supclust), as shown in Supplementary Figure S1. Subsequently, we patented it (Publication of US20150066379A1). The previously reported IRSN-23 was directly adapted to evaluate the accuracy of Affymetrix microarray. When adapting the microarrays to Affymetrix X3P, Agilent, and Illumina, 16 of the 19 genes in the IRSN-23 were supported. Therefore, we adapted IRSN-23 coefficients recalculated within the training set with 16 genes. The expression data obtained from the raw data were mean-centered, and Z-scaling was adapted for datasets for which the raw data were unavailable. The IRSN-23 IS was calculated by Z-scaling as a pre-processing step by aggregating the expression data of 23 probes to 19 genes to fit different expression platforms such as RNA-Seq, where expression data are calculated on a per-gene basis. Pearson’s correlation coefficient between the IRSN-23 score (23 probes) as the original model and the IRSN-23 score (19 genes) as the IS in the OUH Internal Validation (n = 59) used in a previous report was 0.99.

#### TMB and HRD

TMB was calculated as the ratio of the total number of mutations detected to the total size of the coding region (SureSelect Human All Exon V6, Agilent Technologies, 357 MB) [17]. The HRD score was determined as the sum of three factors (NtAI, LST, and HRD-LOH) [17].

#### Gene signatures

The OncotypDx RS Code for Affymetrix microarrays was obtained from the ‘genefu’ R package (https://github.com/bhklab/genefu). PAM50 was also employed to classify breast cancer as luminal A (LumA), luminal B (LumB), HER2-enriched (HER2), or basal-like (basal) following a previously described method [18]. Tumors were classified into clusters closest to each subtype, with the exception of the normal breast-like subtype. To compare with immune-related genes, the gene expression levels of IGG (a 14-gene immunoglobulin B-cell) signature [19], PD-L1, and PD-1 were analyzed after Z-scaling for each dataset. The IGG signature was calculated as the sum of the expression levels of 11 genes (CD27, CD79A, HLA-C, IGJ, IGKC, IL2RG, CXCL8, LAX1, NTN3, PIM2, and TNFRSF17) listed on the Affymetrix HGU-133 Plus 2.0 platform. The expression of PD-L1 was determined as the sum of the expression levels for two probes, 223834_at and 227458_at, while the expression of PD-1 was assessed using the probe 207634_at. All probe IDs correspond to the Affymetrix HGU-133 Plus 2.0 platform.

### Immunohistological examination

Immunohistochemical assays for ER and progesterone receptor (PR) were conducted as previously described, with cutoff values of 1% for ER and PR [16]. HER2 expression was analyzed via fluorescence in situ hybridization according to the ASCO/CAP 2018 guidelines [20], and TILs were evaluated using hematoxylin and eosin-stained sections according to the International TILs Working Group 2014 [21]. TILs of > 20% was defined as high and that of < 20% as low. Tumors were classified into four subtypes: ER + HER2−, ER( +), and HER2(−); ER + HER2 + , ER( +), and HER2( +); ER−HER2 + , ER(−), and HER2( +); and ER−HER2−, ER(−), and HER2(−). pCR for NAC was defined as the complete disappearance of invasive tumor cells in the breast regardless of the presence or absence of ductal carcinoma in situ in the breast and a negative lymph node.

### Statistical analysis

All statistical analyses were conducted using R software version 3.6.3. The association between various parameters was evaluated using the chi-square or Fisher’s exact test. The DeLong test was used to compare the area under the curve (AUCs). All tests were two-tailed, and P < 0.05 indicated statistical significance. Pearson’s correlation coefficients and the averaging method were employed for unsupervised cluster analysis, and the results are shown as heat maps. Gene set enrichment analysis (GSEA) was performed on genes with principal component loadings greater than 0.10 in absolute value. GSEA was performed using DAVID Functional Annotation Bioinformatics (https://david.ncifcrf.gov/). Kaplan–Meier (KM) survival analysis for distant recurrence-free survival for each breast cancer subtype was also performed. The log-rank test was used to compare the KM curves.

## Results

### Reproducibility

#### New validation datasets without anti-HER2 therapy using Affymetrix Human Genome U133 Arrays

The IRSN-23 signature was applied to several independent datasets to classify breast cancer patients into Gp-R (good responders) or Gp-NR (non-responders). In the OUH new validation set (n = 112), 38% of patients were classified as Gp-R (n = 42), and 63% as Gp-NR (n = 70). Within the ER + HER2- subgroup (n = 74), only four patients achieved pCR. In total, 13 patients (11.6%) in the OUH dataset achieved pCR without anti-HER2 therapy, with a significantly higher rate in the Gp-R group compared to the Gp-NR group (29% vs. 1%, P = 2.35E−05, Supplementary Figure S2A, Supplementary Table S1).

Similar results were observed in other datasets, further supporting the predictive accuracy of IRSN-23. In the GSE25066 dataset (n = 488), the pCR rate was significantly higher in the Gp-R group (38%) compared to the Gp-NR group (10%; P = 8.39E-13, Supplementary Figure S2B). Similarly, in GSE28844 (n = 32), the Gp-R group achieved a pCR rate of 29%, while no patients in the Gp-NR group achieved pCR (P = 0.028, Supplementary Figure S2C). In the GSE42822 dataset (n = 66), the Gp-R group demonstrated a higher pCR rate (65%) compared to the Gp-NR group (26%; P = 6.55E−03, Supplementary Figure S2D). Consistent with these findings, the GSE140494 dataset (n = 91) also showed that the pCR rate was higher in the Gp-R group (39%) than in the Gp-NR group (16%; P = 0.03, Supplementary Figure S2E). These results underscore the robust predictive capability of IRSN-23 across diverse datasets.

#### New external validation datasets without anti-HER2 therapy using different microarrays

To validate the reproducibility of IRSN-23 across various platforms, we applied the signature to additional microarray datasets. In GSE4779 (Affymetrix Human X3P arrays, n = 102), Gp-R had a significantly higher pCR rate than Gp-NR (54% vs. 24%, P = 3.53E−03, Supplementary Figure S3A). Agilent data (GSE21974, n = 32) and Illumina data (GSE34138, n = 178) confirmed these findings, with Gp-R showing higher pCR rates than Gp-NR in both cohorts (P = 3.22E−03 and P = 3.63E−05, respectively; Supplementary Figures S3B, C, Supplementary Table S1).

#### Datasets of NAC with anti-HER2 therapy

In patients receiving NAC combined with anti-HER2 therapy, there were no statistically significant differences in pCR rates between Gp-R and Gp-NR in several datasets. In the OUH cohort (n = 34), pCR rates were 78% for Gp-R and 63% for Gp-NR (P = 0.46, Supplementary Figure S4A). In GSE37946 (n = 50), pCR rates were 64% for Gp-R and 50% for Gp-NR (P = 0.55, Supplementary Figure S4B). In GSE42822 (n = 25), Gp-R showed a lower pCR rate (14%) than Gp-NR (61%, P = 0.073, Supplementary Figure S4C). In GSE66399 (n = 88), Gp-R had a 43% pCR rate versus 23% for Gp-NR (P = 0.059, Supplementary Figure S4D). In GSE130788 (n = 110), there was no significant difference in pCR between Gp-R and Gp-NR (52% vs. 35%, P = 0.084) in pre-treatment samples. However, when IRSN-23 was reassessed two weeks post-treatment, Gp-R showed a significantly higher pCR rate than Gp-NR (P = 0.025, Supplementary Figures S4E, F, Supplementary Table S1).

These results suggest that IRSN-23 can capture dynamic changes in the immune microenvironment during anti-HER2 therapy.

#### Pooled analysis and meta-analyses without anti-HER2 therapy

Pooled analysis of independent validation datasets and public datasets without anti-HER2 therapy demonstrated significantly higher pCR rates in the Gp-R group (40%, 170/424) compared to the Gp-NR group (12%, 83/679, P = 2.02E−26, Supplementary Figure S5A, Supplementary Table S1). In patients treated with anti-HER2 therapy, pCR rates were also significantly higher in Gp-R (49%, 61/120) compared to Gp-NR (35%, 64/184, P = 0.017, Supplementary Figure S5B).

**Fig. 1:**
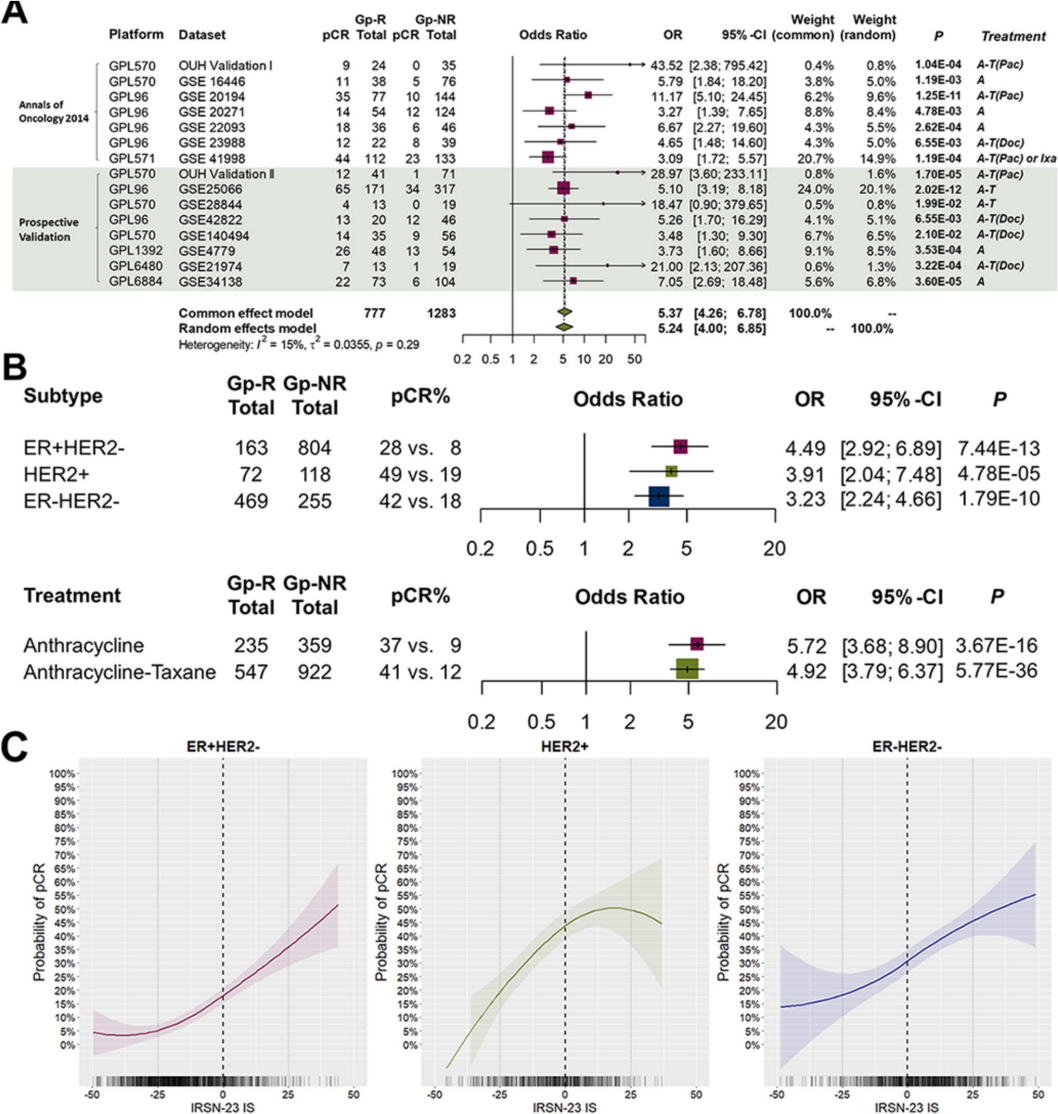
Overview of IRSN-23 pCR-prediction without anti-HER2 therapy in breast cancer. **A** Meta-analysis of all datasets. The gray-shaded areas show the results of the present study. **B** Pooled analysis of each subtype and treatment. **C** IRSN-23 immune scores (IS) and pCR rates for each subtype. The solid lines and bands represent loess regression fitting (span = 2), and 95% confidence intervals were implemented using the R function geom_smooth. Abbreviations in the figure are as follows: *GPL570* Affymetrix Human Genome U133 Plus 2.0 Array, *GPL571* Affymetrix Human Genome U133A 2.0 Array, *GPL96* Affymetrix Human Genome U133A Array, *GPL6884* Illumina HumanWG-6 v3.0 expression beadchip, *GPL1352* Affymetrix Human X3P, *GPL6480* Agilent-014850 Whole Human Genome Microarray 4 × 44 K G4112F, *A* anthracycline, *T* taxane, *Pac* paclitaxel, *Doc* docetaxel, *Ixa* ixabepilone. Predictors of chemotherapy sensitivity and clinical utility

A meta-analysis combining all datasets from previous and current studies confirmed that Gp-R was a significant predictor of pCR across all breast cancer subtypes (OR = 5.24, 95% CI 4.00–6.85, Fig. 1A). Subtype-specific analyses indicated that Gp-R was more likely to achieve pCR across ER + HER2−, ER ± HER2 + , and ER−HER2− breast cancer subtypes, as well as with anthracycline and anthracycline-taxane therapies (odds ratios ranging from 3.23 to 5.72, Fig. 1B).

#### Predictors of chemotherapy sensitivity

**Fig. 2:**
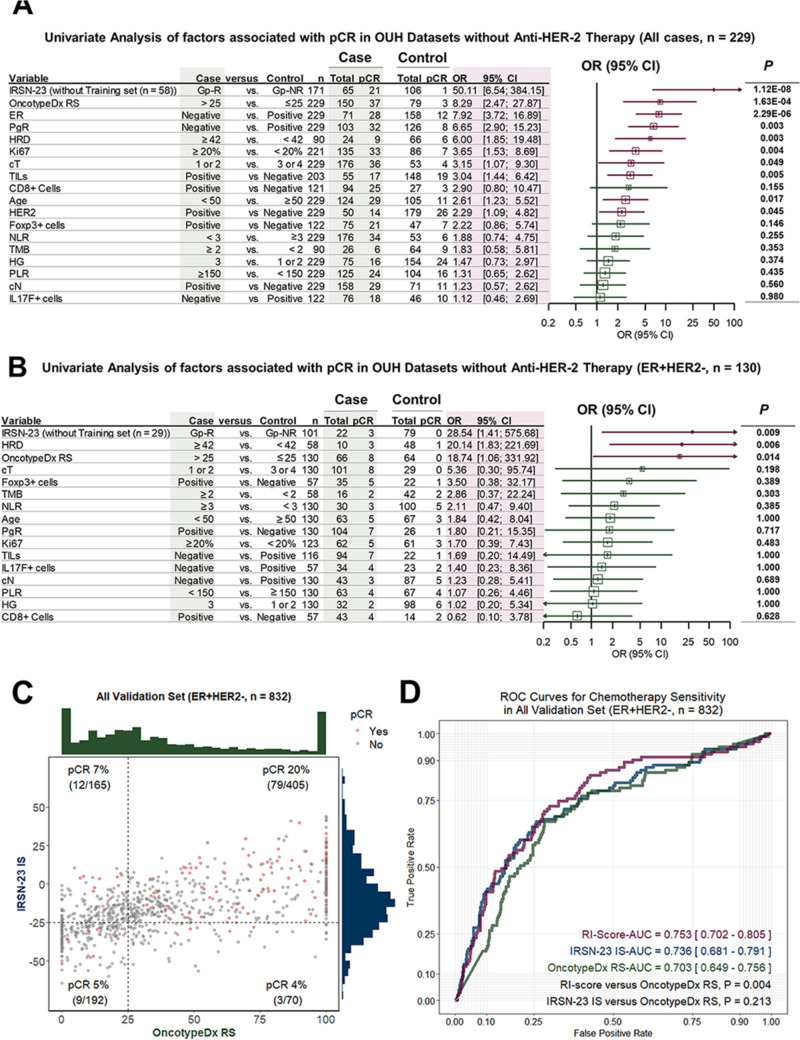
pCR predictors and collaboration with IRSN-23 and Oncotype Dx. The forest plot shows the univariate analyses of variables related to pCR for **A** all cases and **B** ER + HER2− in the OUH datasets. **C** Dot plot and **D** ROC curves illustrating the relationship between IRSN-23 IS, Oncotype Dx RS, Oncotype Dx RS + IRSN-23 IS (RI-score), and pCR in ER + HER2− across all the previous and current validation sets. *cT* clinical tumor size, *cN* clinical nodal status, *ER* estrogen receptor, *PgR* progesterone receptor, *HER2* human epidermal growth factor receptor 2, *HG* histological grade, *TMB* tumor mutation burden, *HRD* homologous recombination deficiency, *TIL* tumor-infiltrating lymphocytes, *NLR* neutrophile-lymphocyte ratio, *PLR* platelet- to-lymphocyte ratio

Univariate analysis in the OUH dataset without trastuzumab (n = 229) identified several factors significantly associated with pCR, including IRSN-23 Gp-R (OR = 50.11, P = 1.12E−08), Oncotype Dx RS > 25 (OR = 8.29, P = 1.63E−04), ER-negative status (OR = 7.92, P = 1.34E−08), PgR-negative status (OR = 6.65, P = 2.29E−06), HRD ≥ 42 (OR = 6.00, P = 0.003), Ki67 ≥ 20% (OR = 3.65, P = 0.004), and TILs-positive (OR = 3.04, P = 0.005, Fig. 2A).

In ER + HER2− breast cancer, IRSN-23 Gp-R (OR = 28.54, P = 0.009), HRD ≥ 42 (OR = 20.14, P = 0.006), and Oncotype Dx RS > 25 (OR = 18.74, P = 0.014) were all significantly associated with pCR (Fig. 2B).

A combined analysis of Oncotype Dx RS and IRSN-23 IS showed that the most NAC-sensitive group was IS > −25 + RS > 25, with a 20% pCR rate. This was followed by IS > −25 + RS < 25 (7%), IS ≤ −25 + RS ≤ 25 (5%), and IS ≤ −25 + RS > 25 (4%, P = 3.28E−03, Fig. 2C). The combined recurrence and immune score (RI-Score, Supplementary Figure S6) showed superior predictive accuracy for pCR compared to Oncotype Dx RS alone (AUC = 0.753 vs. 0.703, P = 0.004, Fig. 2D).

We conducted a detailed analysis of immune factors and their relationship with chemotherapy sensitivity. The 14-gene immunoglobulin B-cell signature, referred to as the IGG signature, was included to evaluate its predictive utility as an immune-related marker. Notably, there was no overlap between the genes in the IGG signature and those in IRSN-23. Additional comparisons were made with TILs, PD-L1, and PD-1. In the OUH validation datasets (excluding the IRSN-23 training set), IRSN-23 IS showed the highest predictive performance for chemotherapy sensitivity, achieving an AUC of 0.767 on the ROC curve. The IGG signature followed with an AUC of 0.684. Interestingly, the IGG signature demonstrated particularly high predictive accuracy within the range of IRSN-23 IS > 25, suggesting that B-cell-related genes could play a role in enhancing predictive precision (Supplementary Figures S7). Additionally, we assessed the correlation between the expression levels of IRSN-23 and other immune factors, including IGG, TILs, PD-L1, and PD-1. Among these, a strong correlation was observed between IRSN-23 and PD-L1 (correlation coefficient r = 0.751), despite PD-L1 not being included among the genes that comprise IRSN-23 (Supplementary Figures S8). This novel observation underscores the potential immunological mechanisms underlying IRSN-23’s predictive capability.

### Characterization of IRSN-23 and its impact on breast cancer subtype classification

We further analyzed the association between IRSN-23 Gp-R and clinicopathological factors. Significant correlations were found with age > 50 years (P = 1.37E−04), high tumor grade (P = 0.002), Ki67 ≥ 20% (P = 8.64E−05), ER-negative status (P = 1.93E−13), and PgR-negative status (P = 2.99E−10). TILs-positive was associated with CD8 + (P = 0.037) and Foxp3 + regulatory T cells (P = 0.012), but not with blood markers like NLR (P = 0.425) or PLR (P = 0.803). Tumor mutational burden (TMB ≥ 2, P = 5.26E−04) and HRD ≥ 42 (P = 0.009) were also significantly associated with Gp-R (Supplementary Figure S9).

**Fig. 3:**
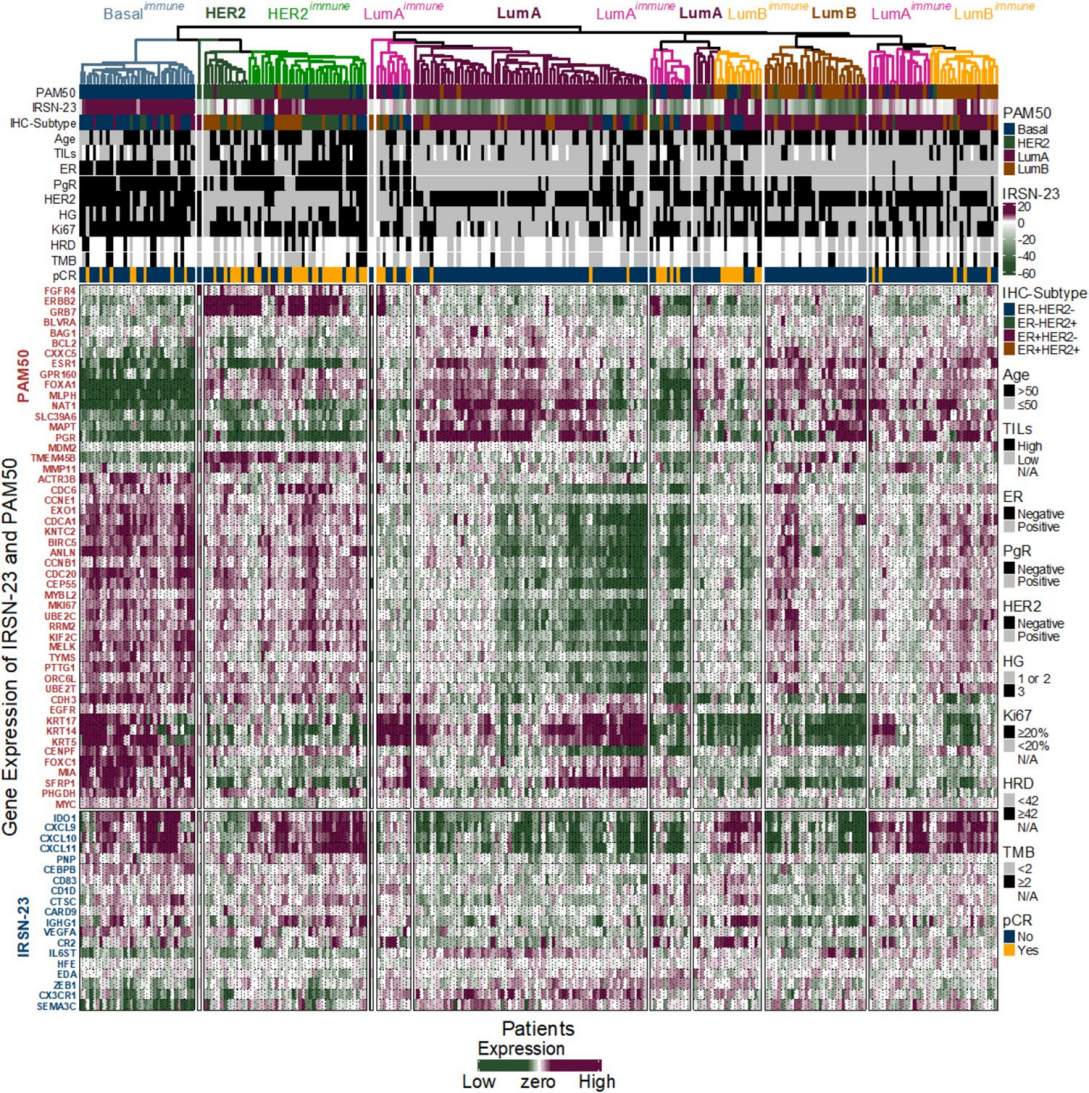
Unsupervised clustering by PAMIR (PAM50 and IRSN-23) genes in OUH datasets (n = 263). *ER* estrogen receptor, *PgR* progesterone receptor, *HER2* human epidermal growth factor receptor 2, *HG* histological grade, *TMB* tumor mutation burden, *HRD* homologous recombination deficiency, *TIL* tumor-infiltrating lymphocytes, *pCR* pathological complete response

Unsupervised clustering with PAM50 and IRSN-23 genes (PAMIR, Fig. 3) identified novel immune-enriched subgroups such as LumA^immune^, LumB^immune^, HER2^immune^, and Basal^immune^ within established breast cancer subtypes compared to PAM50 alone (Supplementary Figure S10). Moreover, the PAMIR revealed potential subdivisions within LumA^immune^ and LumB^immune^, driven by differences in HRD and TMB statuses as well as expression levels of genes such as PGR, KRT14, and KRT17. These findings suggest the possibility of refining breast cancer subtypes in future studies based on both intrinsic tumor characteristics and immune responses.

**Fig. 4:**
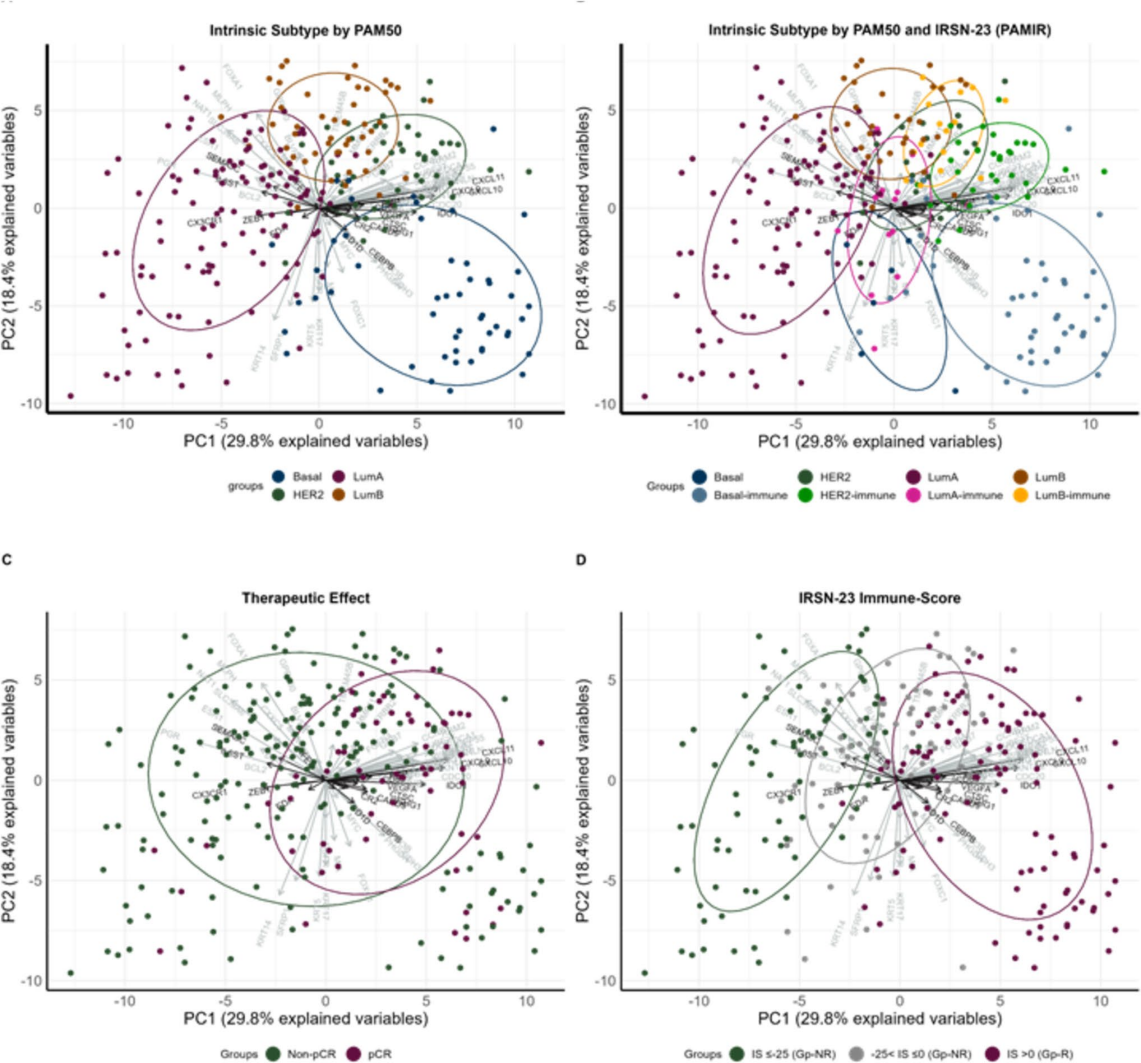
Principal component (PC) analysis by PAMIR (PAM50 and IRSN-23) genes in OUH dataset (n = 263). The x-axis (first principal component), y-axis (second principal component), and dot placement in Figures A-D are all the same. The IRSN-23 and PAM50 genes are shown in black and grey, respectively. Each circle represents a range of 66% of each group’s distribution. The dots are labeled and color-coded by **A** intrinsic subtype by PAM50, **B** intrinsic subtype by PAMIR, **C** therapeutic effect, and **D** IRSN-23 immune score (IS), respectively

Principal component analysis (PCA) of PAMIR gene expression (Fig. 4A, B) revealed that the immune-related pathways (e.g., cytokine-cytokine receptor interactions and chemokine signaling pathways) were enriched in the first principal component (Supplementary Table S2) compared to PAM50 alone (Supplementary Figure S11), which predominantly involved cell cycle and estrogen signaling pathways (Supplementary Table S3). This further underscores the importance of integrating immune responses into breast cancer classification frameworks.

**Fig. 5:**
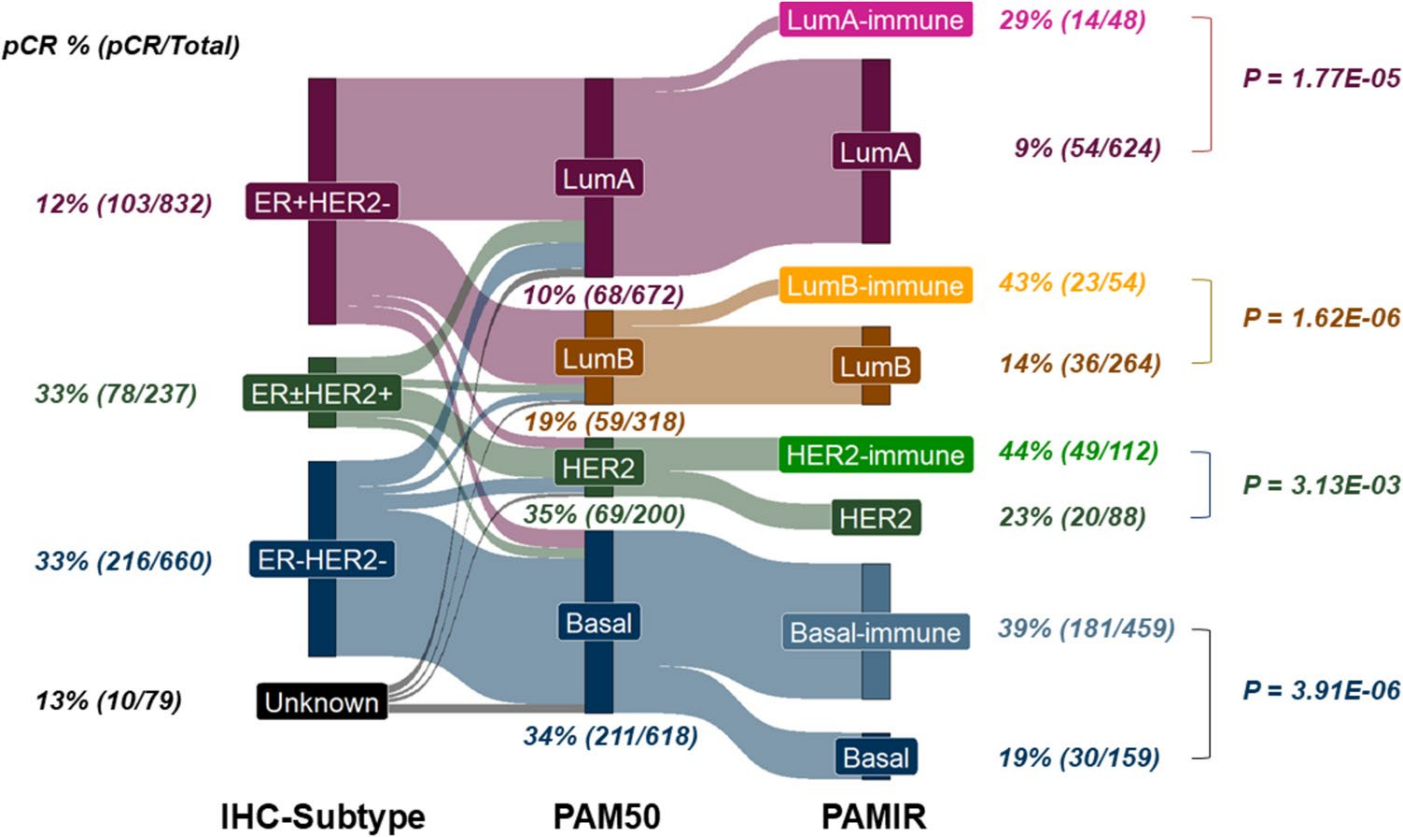
Evolution of breast cancer classifications and its relationship to pCR. Sankey plot showing the association between breast cancer classifications and the pCR using all the previous and current Affymetrix Human Genome U133 array datasets without anti-HER2 therapy (n = 1808)

PAMIR-based classification also provided clearer differentiation of treatment-sensitive subgroups, as evidenced by a rightward shift of chemotherapy-sensitive groups in Fig. 4C. IRSN-23 genes were predicted to surround these groups, further supporting its role in refining breast cancer subtyping based on immune factors. Importantly, the PAMIR classification demonstrated the ability to separate all PAM50 subtypes into chemotherapy high- and low-sensitivity groups (Fig. 5, Supplementary Figure S12).

#### Prognosis after NAC

Prognostic analysis after neoadjuvant chemotherapy (NAC) was conducted for 769 patients from the OUH, GSE16446, and GSE25066 datasets. The median follow-up was 44 months, during which 174 distant recurrence events were recorded. Using the PAMIR classification, we grouped LumA and LumB as LumA/B, and their immune-enriched counterparts as LumA/B^immune^. Similarly, HER2 and HER2^immune^, as well as Basal and Basal^immune^, were analyzed individually.

**Fig. 6:**
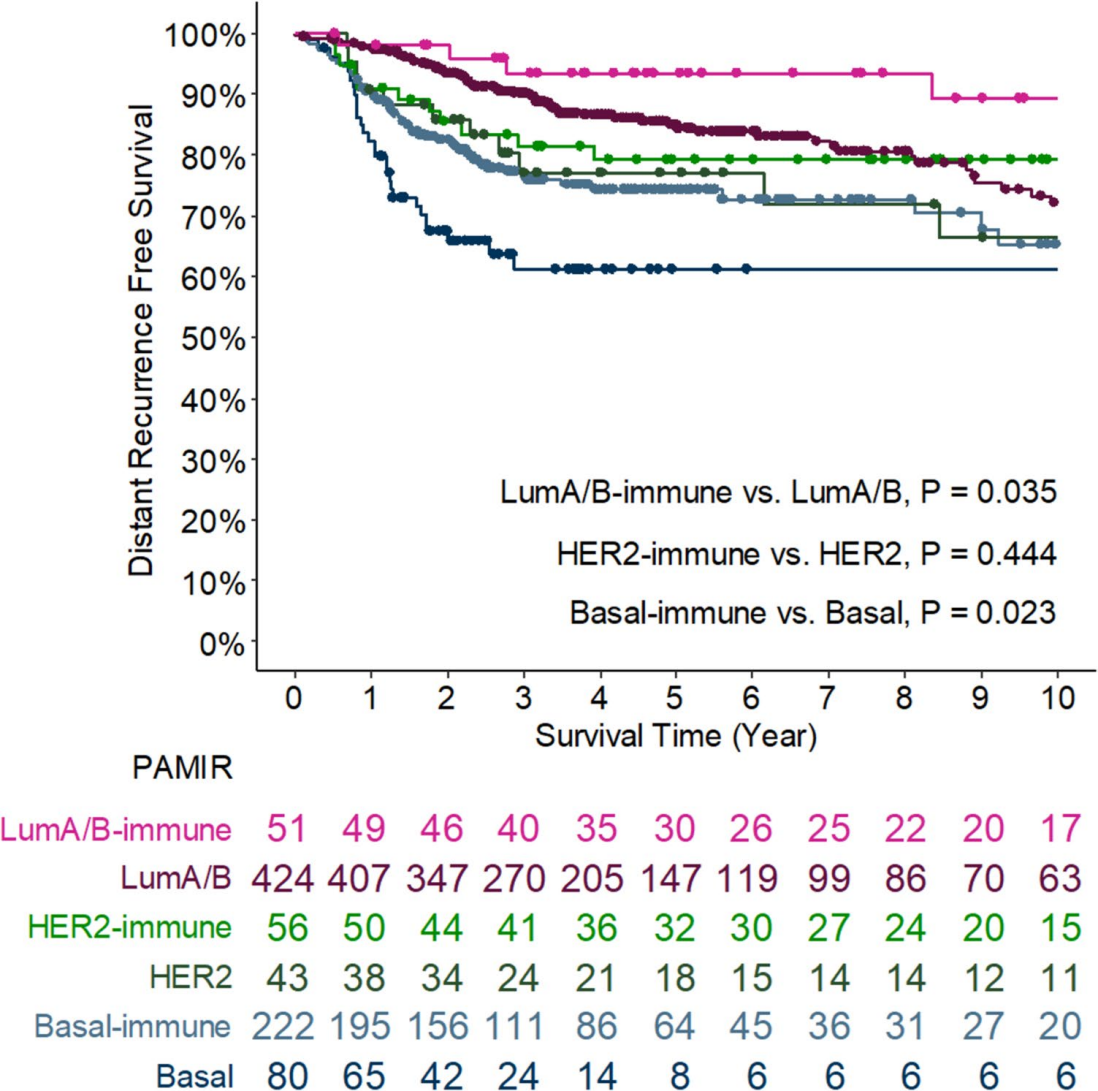
PAMIR (PAM50 and IRSN-23) subtype classification and disease-free survival after neoadjuvant chemotherapy

The results showed that Basal^immune^ and LumA/B^immune^ groups had significantly better distant recurrence-free survival (DRFS) compared to their non-immune counterparts (Basal and LumA/B). Specifically, Basal^immune^ and LumA/B^immune^ demonstrated improved DRFS compared to Basal and LumA/B, with statistical significance (P = 0.023 and P = 0.035, respectively; Fig. 6). We also conducted an analysis demonstrating that IRSN-23 can stratify non-pCR patients with the Luminal subtype into distinct prognostic subgroups (P = 0.045, Supplementary Figure S13). These results suggest that IRSN-23 can be used not only for predicting chemotherapy response but also for stratifying breast cancer patients based on their prognosis after NAC.

## Discussion

Chemotherapy sensitivity prediction models have been extensively developed; however, few have undergone prospective validation. Among these, DLDA30 has been prospectively evaluated, demonstrating predictive ability for weekly paclitaxel and T/FAC regimens, although its accuracy was limited for FAC, influenced by the treatment regimen [12]. Nearly a decade ago, we introduced the IRSN-23 model. In 2018, the predictive accuracy of IRSN-23 was prospectively validated in the phase 3 TEX trial, which evaluated the efficacy of epirubicin and paclitaxel with or without capecitabine in recurrent breast cancer. Foukakis et al. compared IRSN-23 with other diagnostic models, including ESR1, PIK3CA, and TP53, and reported a linear correlation between IRSN-23 and the four-month tumor shrinkage rate at metastatic sites, particularly in ER-positive, luminal-type breast cancer [22]. In addition to IRSN-23, other immune-related gene signatures have shown promise in predicting treatment outcomes. Notably, the IGG signature, an immune-related gene set, has recently been reported to be associated with improved prognosis and pathological complete response (pCR) in early-stage triple-negative breast cancer [19]. These advances underscore the growing importance of integrating immune-related markers into chemotherapy sensitivity prediction models to enhance their clinical utility.

The reproducibility and new validation of chemotherapy sensitivity prediction models are essential for clinical utility. In this study, IRSN-23 demonstrated robust predictive performance. These findings underscore the reproducibility of IRSN-23 in predicting chemotherapy sensitivity without anti-HER2 therapy, reinforcing its potential clinical applicability across a diverse range of patient profiles and treatment regimens. The new external validation of IRSN-23 using different microarray platforms further bolstered the model’s reliability. The consistent results across different platforms, including Affymetrix, Agilent, and Illumina, highlight the robustness of IRSN-23 and its adaptability to varying technological frameworks, a critical feature for clinical implementation. In conjunction with this study, we tested the transfer of IRSN-23 from microarray to RNA-Seq. IRSN-23 exhibited a highly significant correlation coefficient (Pearson correlation coefficient r = 0.98) in microarray and RNA-Seq analyses of the corresponding samples. Only 1 of the 43 cases showed a discrepancy in IRSN-23 diagnosis (Supplementary Figure S14). This high reproducibility may indicate the measurability of the IRSN-23.

Interestingly, when applied to datasets involving patients receiving NAC combined with anti-HER2 therapy, the pooled analysis showed a significant difference, but the predictive performance of IRSN-23 was limited. However, reassessment with IRSN-23 two weeks after treatment initiation revealed significantly improved pCR rates in the Gp-R group, This suggests that IRSN-23 may be more sensitive to dynamic changes in the tumor microenvironment during treatment, particularly in response to anti-HER2 therapies. This finding may be related to antibody-dependent cell-mediated cytotoxicity, a known therapeutic effect of HER2-targeted therapy [23]. Thus, while IRSN-23 may not serve as a reliable baseline predictor in these contexts, its utility could lie in real-time monitoring of treatment response, warranting further investigation into optimal assessment timings to maximize its clinical value. In contrast, the utility of IRSN-23 was more evident in luminal and TNBC cases, where immune involvement plays a prominent role. For instance, in ER + HER2− breast cancer, IRSN-23 Gp-R was strongly associated with pCR (OR = 28.54, P = 0.009). Similarly, in TNBC, where immune activity often predicts treatment outcomes, IRSN-23 has shown promise in stratifying patients based on chemotherapy sensitivity. These findings underscore the need for further research to refine IRSN-23’s application across specific subtypes, with a particular focus on luminal and TNBC, where its predictive and prognostic utility appears strongest.

In recent years, ER + HER2− breast cancers have been determined by Oncotype Dx RS as an indication for chemotherapy based on the risk of recurrence [24]; therefore, collaboration with Oncotype Dx was also evaluated in terms of its clinical utility value. Univariate analyses of predictors of chemotherapy sensitivity revealed that IRSN-23 was among the most significant factors associated with pCR in the OUH datasets. For example, in ER + HER2− breast cancer, IRSN-23 Gp-R (OR = 28.54, P = 0.009), HRD ≥ 42, and Oncotype Dx RS > 25 were all significantly correlated with pCR. A recent report by Edlund et al. explained that high cut-off values are one of the reasons why genetic diagnosis has not been applied clinically [25]. After confirming the reproducibility of the IRSN-23 gene signature, we re-evaluated the previous findings of Gp-R (IRSN-23 IS > 0) and Gp-NR (IRSN-23 IS ≤ 0), we re-evaluated our previous findings, in which we evaluated two values of IRSN-23 IS as continuous values of IRSN-23 IS. This analysis demonstrated that even within the Oncotype DX RS > 25 cohort, IRSN-23 can identify a subgroup with low chemotherapy sensitivity (IS ≤ −25), underscoring its potential to guide treatment decisions for patients who might be overlooked by Oncotype DX alone. Specifically, patients with IS > −25 exhibited a significantly higher pCR rate, whereas those with IS ≤ −25 represented a population less likely to benefit from chemotherapy. Therefore, IRSN-23 serves as a valuable tool for refining chemotherapy indications in hormone receptor-positive breast cancer and may also facilitate the selection of alternative adjuvant therapies, such as CDK4/6 inhibitors or S-1 (oral fluoropyrimidine). This complementary role aligns with current trends in breast cancer treatment, and integrating IRSN-23 into clinical decision-making processes could further enhance personalized treatment strategies.

In terms of breast cancer classification, IRSN-23 offers new insights into tumor subtyping when integrated with PAM50. Our PAMIR classification, which incorporates IRSN-23 with PAM50 genes, further stratifies existing subtypes based on immune-related gene expression. For instance, PAMIR distinguishes between high- and low-immune expression groups (LumA^immune^ vs. LumA) and demonstrates that immune-enriched subtypes (LumA^immune^, LumB^immune^, HER2^immune^, Basal^immune^) have higher pCR rates compared to their non-immune counterparts. This classification highlights the potential of IRSN-23 to redefine breast cancer subtypes, taking into account not only tumor-intrinsic factors but also host immune responses (Supplementary Figure S15). This approach differs from previous classifications, which focused only on the potential nature of the tumor. In 2022, Wolf et al. suggested in their analysis of the I-SPY2 Neoadjuvant Trial (NCT01042379) that breast cancer may form new subgroups based on immune response, supporting our hypothesis [26].

Furthermore, survival analyses indicated that immune-enriched subtypes, such as Basal^immune^ and LumA/B^immune^, had significantly better distant recurrence-free survival (DRFS) than their non-immune counterparts, further supporting the prognostic value of IRSN-23 beyond its predictive utility. Notably, in the non-pCR after NAC, the LumA/B^immune^ subtype was associated with a better prognosis. These findings suggest that IRSN-23 is not only a robust predictor of chemotherapy response but also a potential prognostic marker, particularly when integrated into a broader classification framework like PAMIR.

Finally, this study has several limitations. First, the data registered in public databases vary in terms of breast cancer subtypes and treatment regimens, depending on the original study design. This heterogeneity may affect the generalizability of our findings. Second, the Oncotype Dx RS in this study was calculated based on microarray expression data, which may not be entirely consistent with results obtained from the actual clinical test. Similarly, the IGG signature does not fully correspond to some genes on the microarray platform, potentially leading to slight inconsistencies in its expression values. These factors highlight the need for further validation in standardized and clinically relevant settings.

## Conclusions

IRSN-23 demonstrates reproducible and robust predictive accuracy for chemotherapy sensitivity across a wide range of breast cancer subtypes, especially in non-HER2 therapies. Its adaptability across platforms and the synergy with other biomarkers like Oncotype Dx RS offer promising avenues for improving personalized treatment strategies. Although its baseline predictive power in anti-HER2 therapies is limited, its utility in monitoring treatment response makes it a valuable tool for dynamic clinical assessment. Future studies should focus on further validating IRSN-23 in larger and more diverse patient cohorts and optimizing its integration into breast cancer subtyping frameworks like PAMIR to enhance both its predictive and prognostic applications. These findings hold significant potential for refining breast cancer treatment strategies and improving patient outcomes through more precise and personalized chemotherapy sensitivity predictions.

## Supplementary Information

Below is the link to the electronic supplementary material.

Supplementary file 1 (DOCX 2608 KB)

Supplementary file 2 (DOCX 21 KB)

Supplementary file 3 (DOCX 22 KB)

Supplementary file 4 (DOCX 20 KB)

## Data Availability

The OUH expression data for 263 patients (training set (n = 58), validation set (n = 59), and this new dataset (n = 146)) are available as GSE230881 (https://www.ncbi.nlm.nih.gov/geo/query/acc.cgi?acc=GSE230881) from NCBI’s Gene Expression Omnibus (GEO, http://www.ncbi.nlm.nih.gov/geo/).
