# Supplementary material for: IRSN-23 gene diagnosis enhances breast cancer subtype classification and predicts response to neoadjuvant chemotherapy: new validation analyses
Source: Breast Cancer. 2025 Mar 24;32(3):566–81. doi: 10.1007/s12282-025-01687-6 (PMC11993443; doi:10.1007/s12282-025-01687-6)
Supplement: Supplementary file 1 — (DOCX 2608 KB) [file 12282_2025_1687_MOESM1_ESM.docx]

**
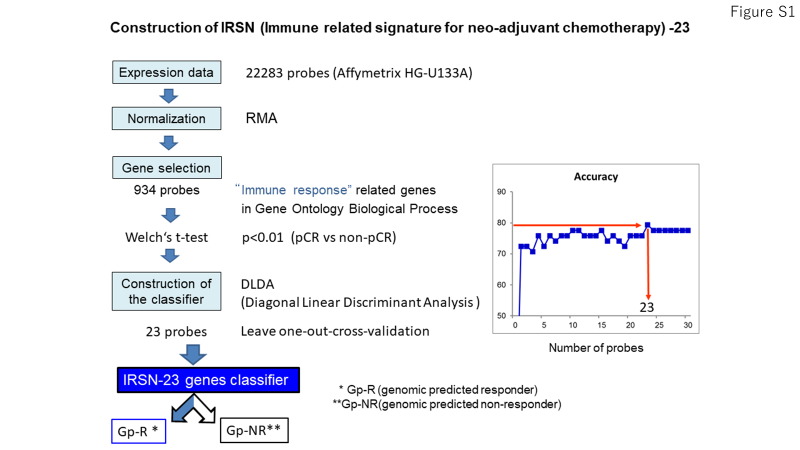
Figure S1. Overview of IRSN-23 Construction**

**Construction of IRSN-23 (Immune-Related Gene Signature for NAC, 23 probes)**

**IRSN-23 genes**

 **Figure S2. Prediction of pCR by IRSN-23 in the prospective external validation datasets without anti-HER2 therapy using Affymetrix DNA microarray**


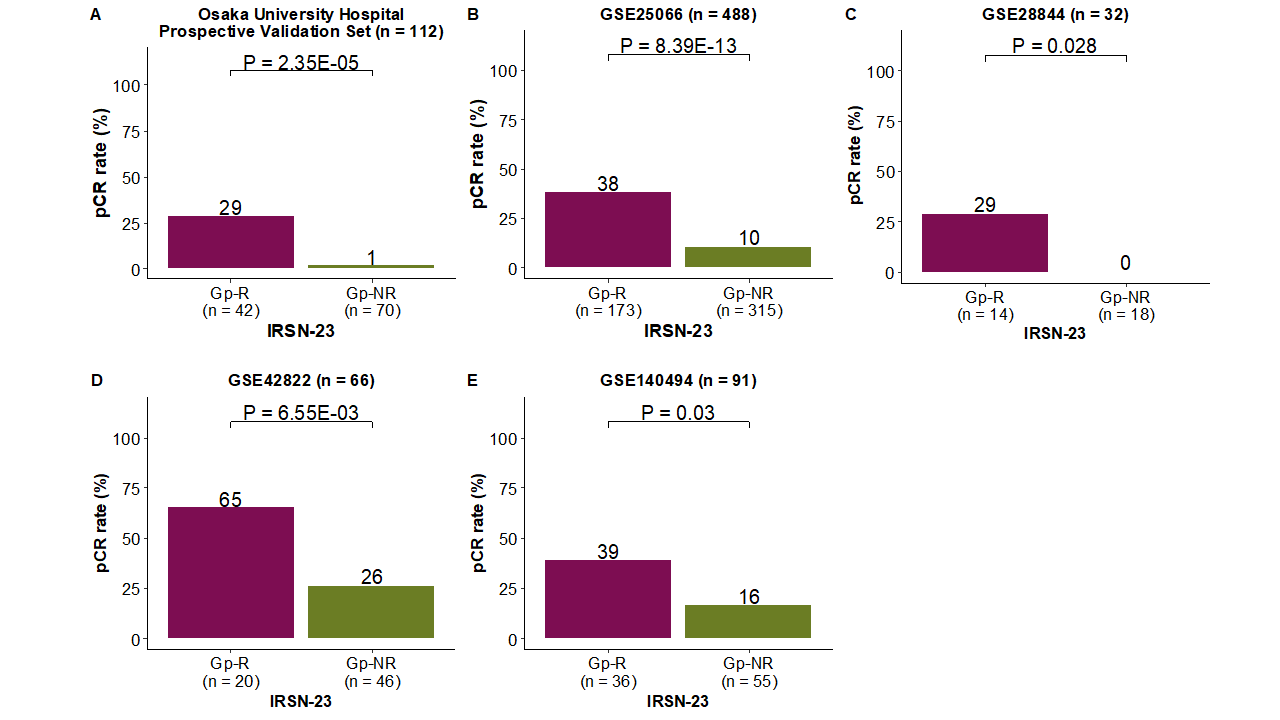
Bar graphs showing a pCR rate (y-axis) and IRSN-23 (x-axis) in (A) Osaka University Hospital prospective validation set, (B) GSE25066, (C) GSE28844, (D) GSE42822, and (E) GSE140494, respectively.

**Figure S3. Prediction by IRSN-23 in the different DNA microarray datasets without anti-HER2 therapy and rectal cancer**

Bar graphs showing a pCR rate (y-axis) and IRSN-23 (x-axis) in (A) GSE4779 Affymetrix X3P array, (B) GSE21974 Agilent, and (C) GSE34138 illumina


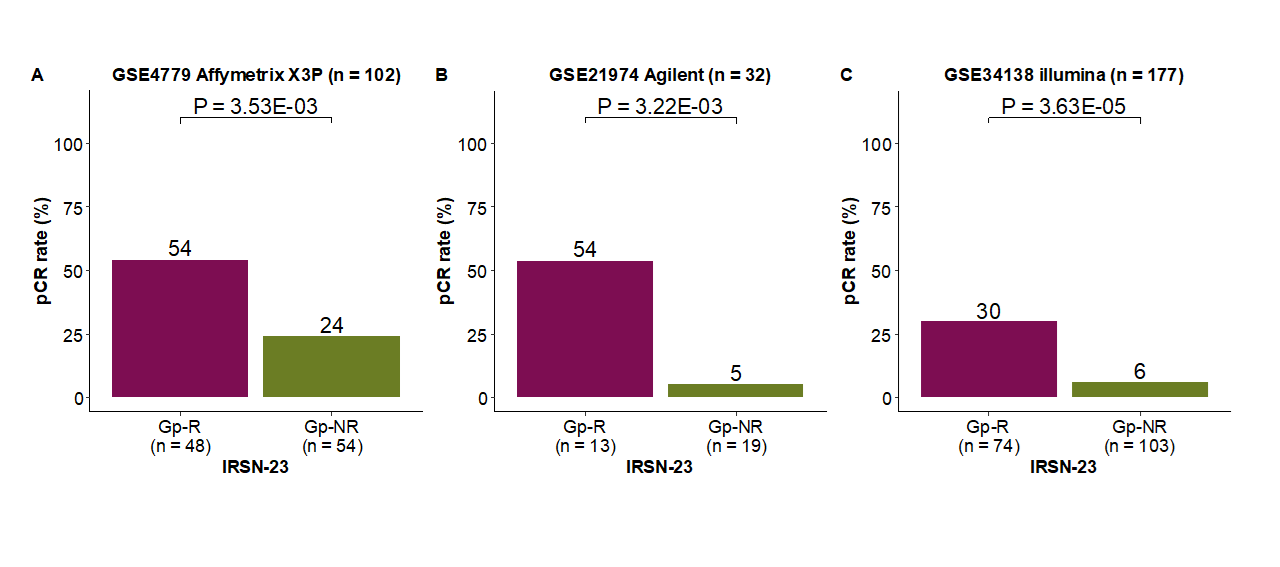


**Figure S4. Prediction of pCR by IRSN-23 in datasets of chemotherapy with anti-HER2 therapy**

Bar graphs showing pCR rate (y-axis) and IRSN-23 (x-axis) in (A) Osaka University Hospital (OUH) prospective validation datasets, (B) GSE37946, (C) GSE42822, (D) GSE66399, (E) GSE130788 baseline only, and (F) GSE130788 integrated analysis of baseline and two weeks after, respectively


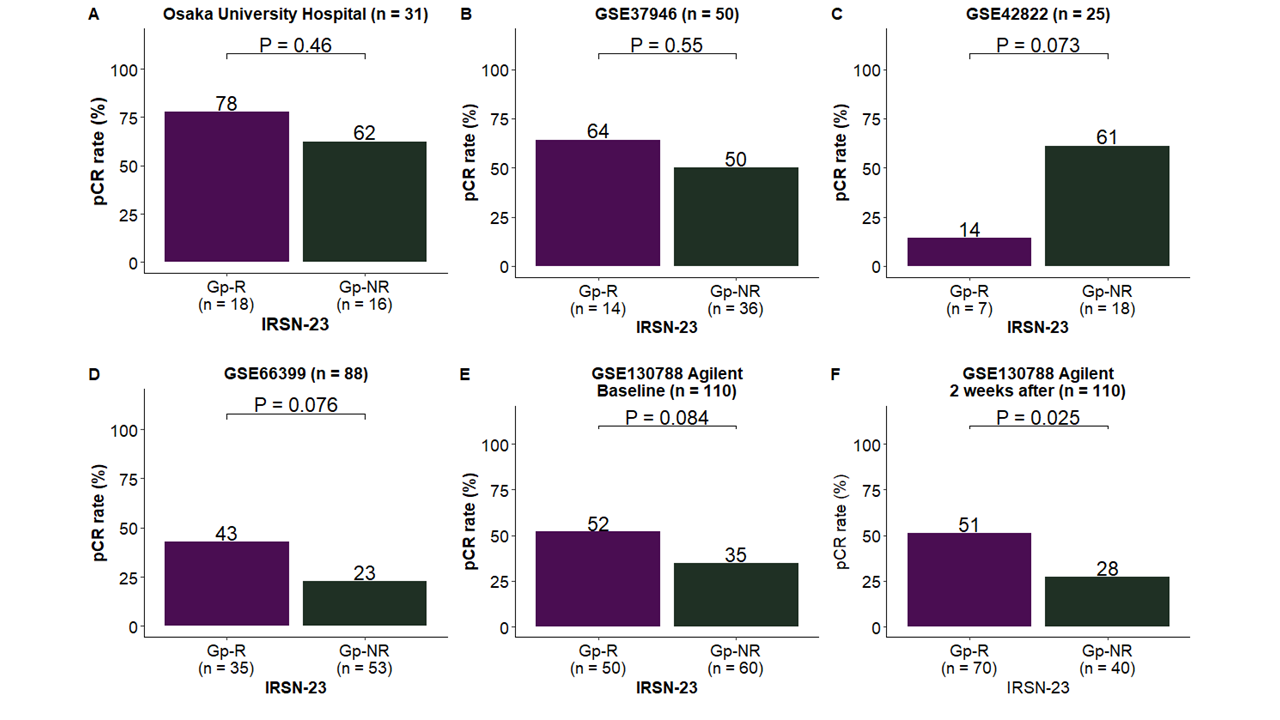


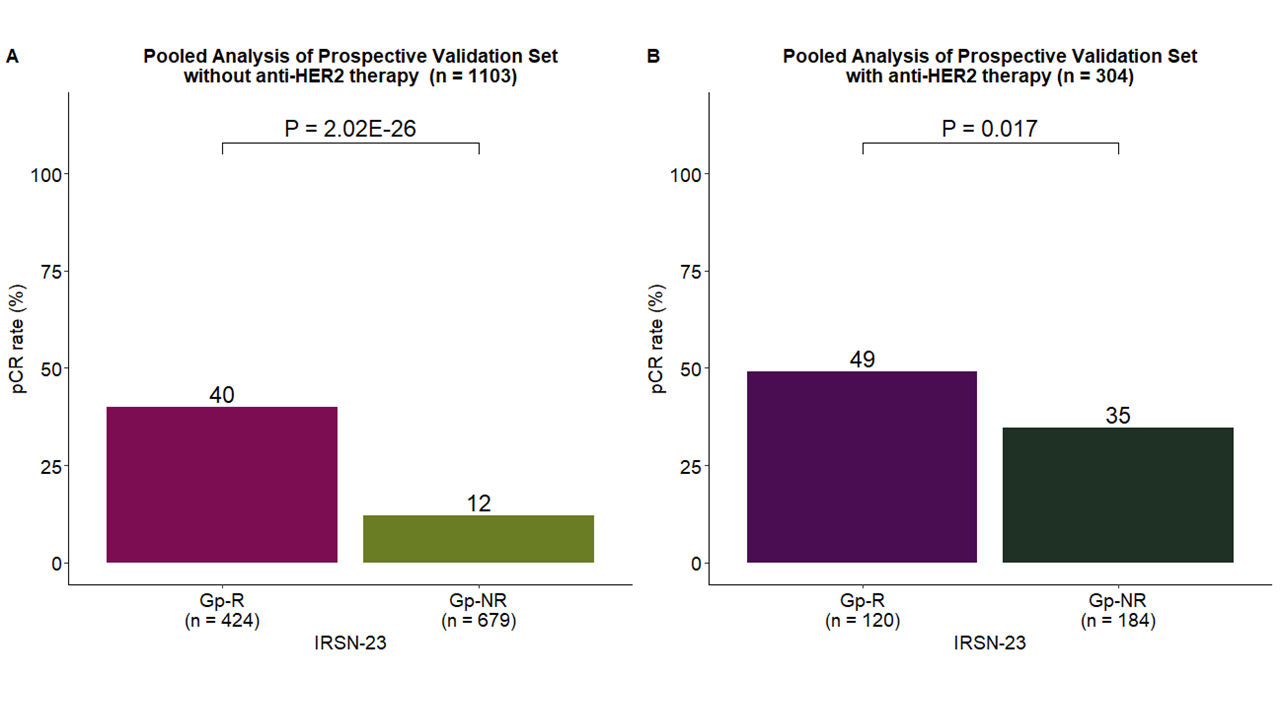
**Figure S5. Pooled analysis of prospective analyses**

**Figure S6. Collaborative model of OncotypeDx RS and IRSN-23 IS to predict chemotherapy sensitivity**


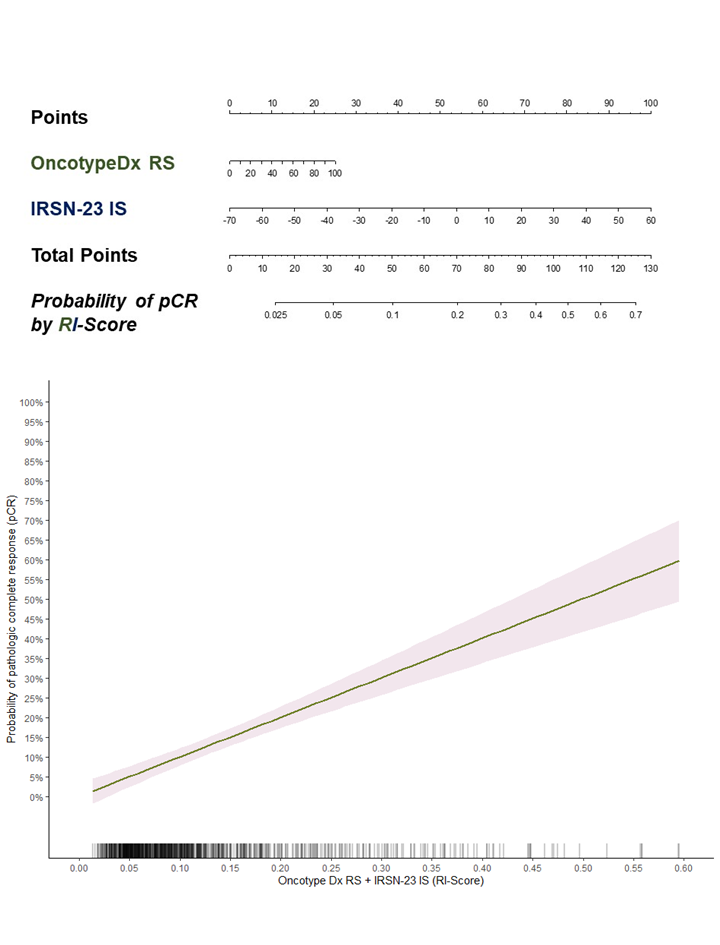


**Figure S7. ROC Curves for Chemotherapy Sensitivity in OUH Validation Sets**

The figure shows ROC curves for pCR and IRSN-23, IGG, TILs, PD-L1, and PD-1, respectively. The ROC curve of IRSN 23 is marked to show the -25, 0, and 25 points of the IRSN 23 scores.

Abbreviation: IGG, 14-gene immunoglobulin B-cell signature; TILs, Tumor-infiltrating lymphocytes; PD-L1, Programmed death ligand 1; PD-1, Programmed cell Death 1

**
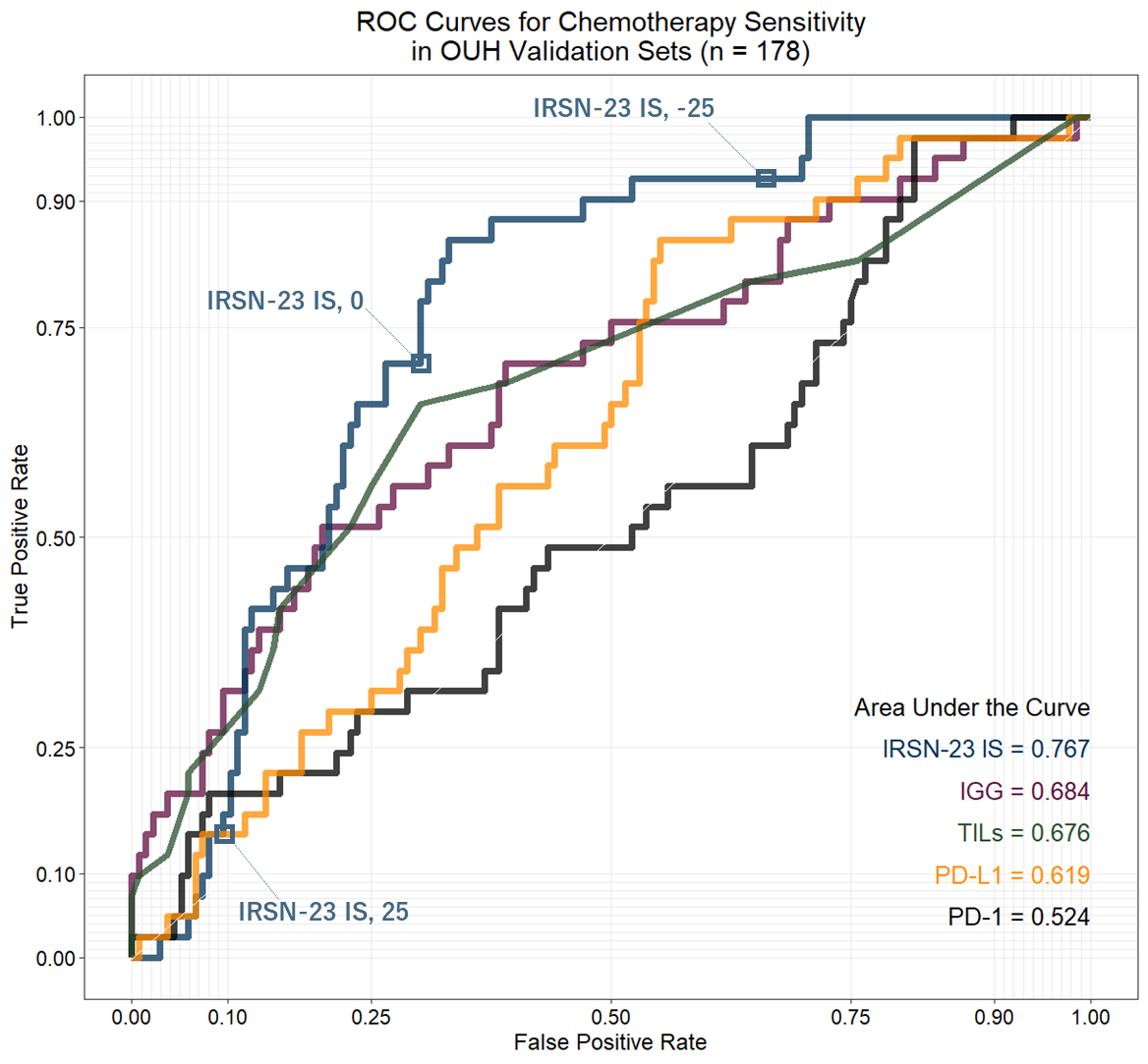
**

**Figure S8. Association of IRSN-23 with Immunological Factors**

The figure shows the relationship between the expression of IGGs(A), TILs(B), PD-L1(C), and PD-1(D) and the IRSN-23 score. Correlation coefficients were calculated using Pearson's correlation coefficient.

Abbreviation: IGG, 14-gene immunoglobulin B-cell signature; TILs, Tumor-infiltrating lymphocytes; PD-L1, Programmed death ligand 1; PD-1, Programmed cell Death 1

**
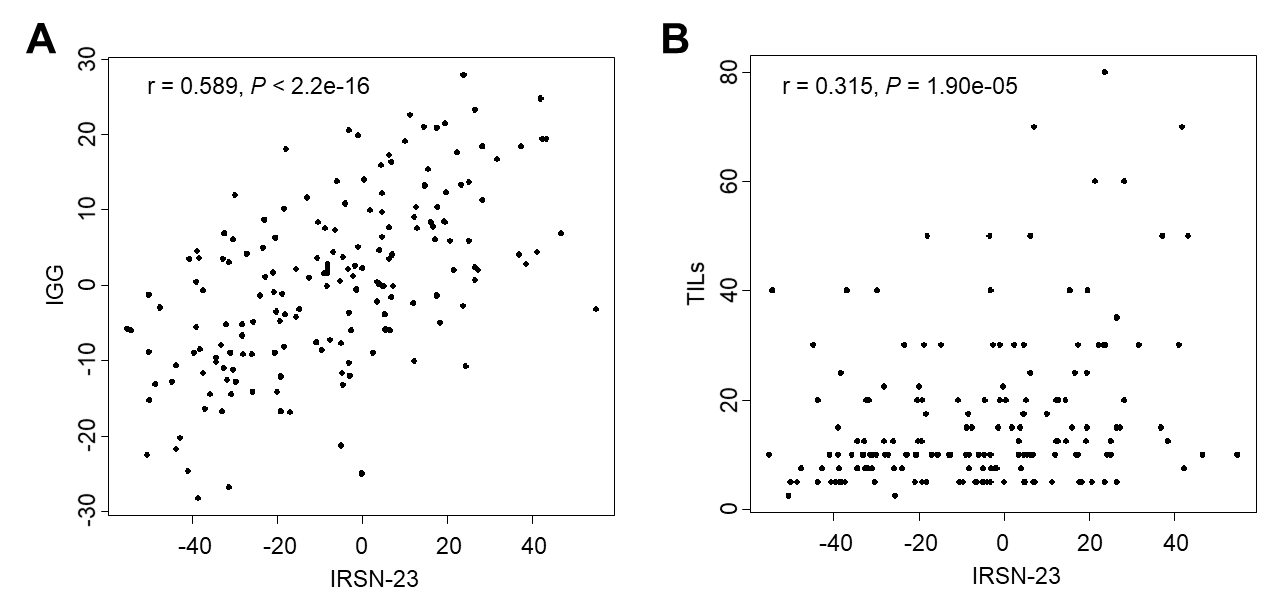
**
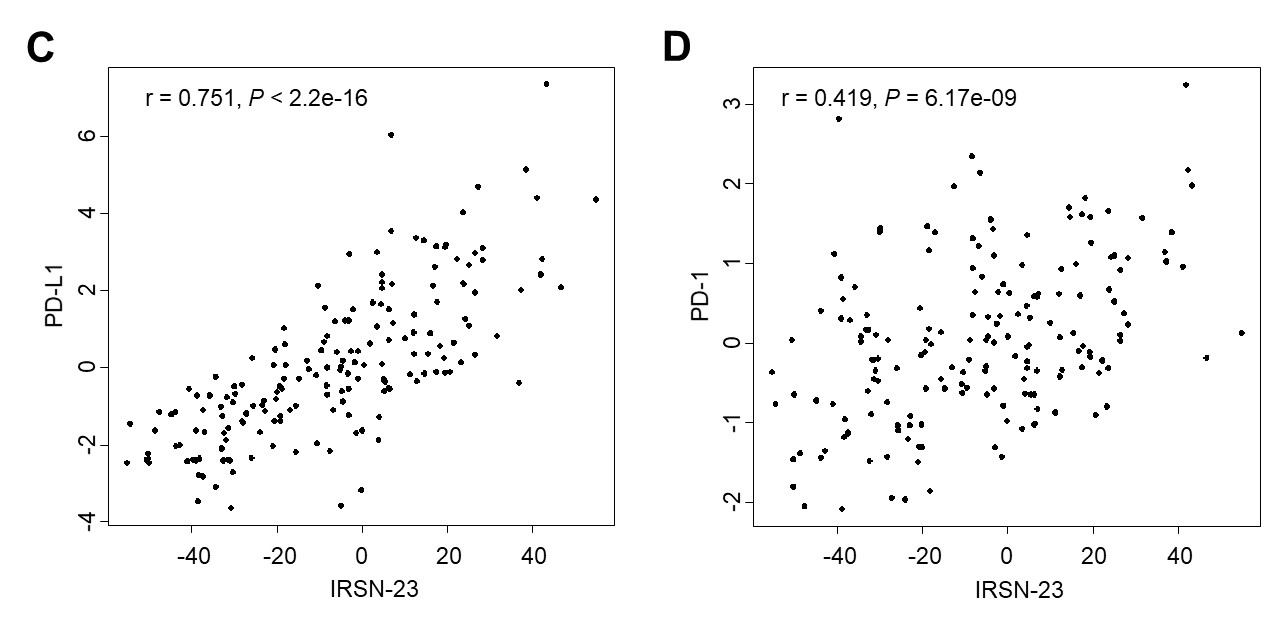


**Figure S9. Relationship between clinicopathological factors and IRSN-23**

The figure shows the univariate analysis for each variable related to the Gp-R of IRSN-23 as the case arm against the control arm.

Abbreviation: cT, clinical Tumor size; cN, clinical nodal status; ER, estrogen receptor, PgR, progesterone receptor; HER2, human epidermal growth factor receptor 2, HG, histological grade; TMB,Tumor mutation burden; HRD, homologous recombination deficiency,TILs, Tumor-infiltrating lymphocytes, NLR, Neutrophile- lymphocyte ratio; PLR, Platelet- to-lymphocyte ratio


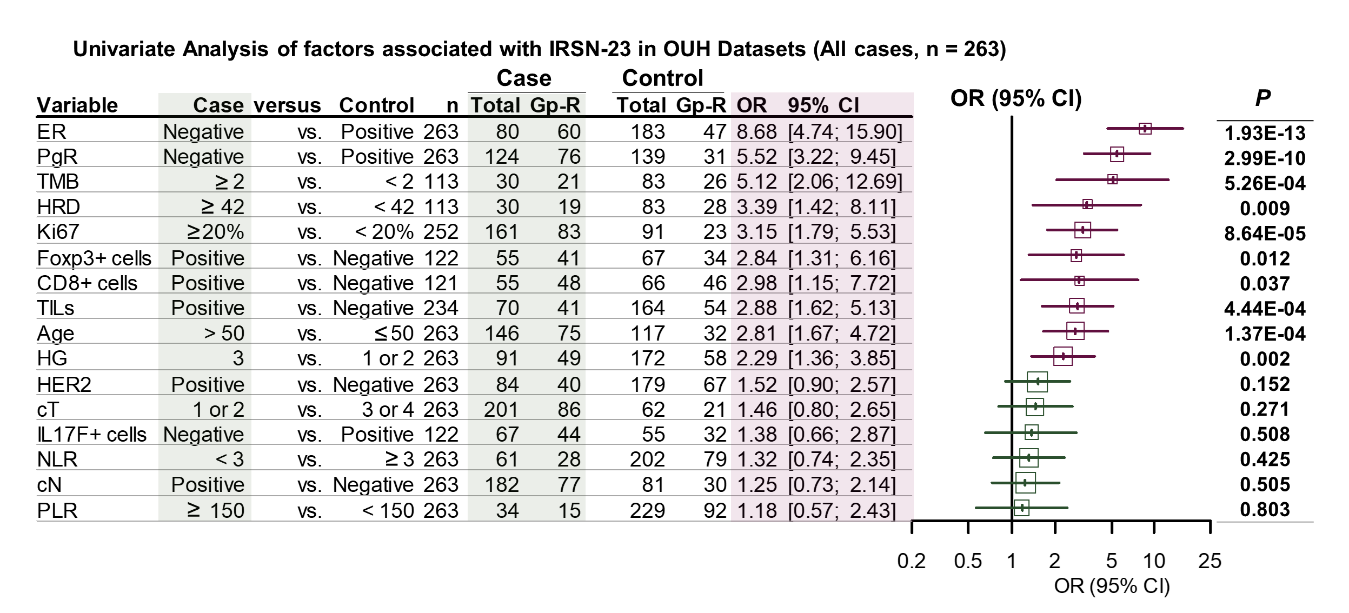


**Figure S10. Unsupervised clustering by PAM50 in OUH datasets (*N* = 263)**

Abbreviation: ER, estrogen receptor; PgR, progesterone receptor; HER2, human epidermal growth factor receptor 2; HG, histological grade; TMB,Tumor mutation burden; HRD, homologous recombination deficiency; TIL, Tumor-infiltrating lymphocytes; pCR, pathological complete response


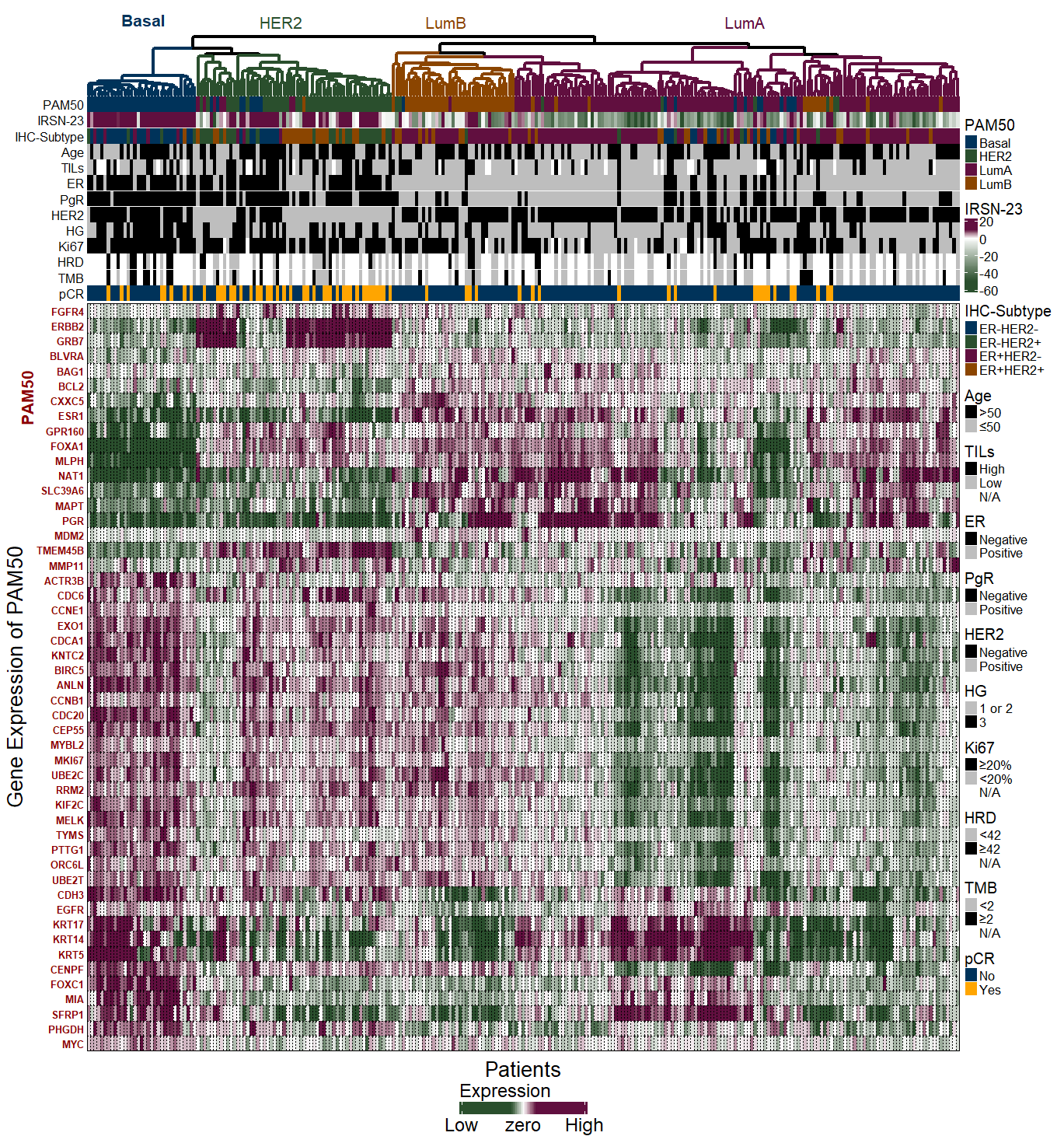


**Figure S11. Principal component (PC) analysis by PAM50 genes in OUH dataset (*N* = 263)**

The x-axis (first principal component), y-axis (second principal component), and dot placement in Figures A-D are all the same. The IRSN-23 and PAM50 genes are shown in black and grey, respectively. Each circle represents a range of 66% of each group’s distribution. The dots are labeled and color-coded by (A) intrinsic subtype by PAM50, (B) intrinsic subtype by PAMIR, (C) therapeutic effect, and (D) IRSN-23 immune score (IS), respectively.


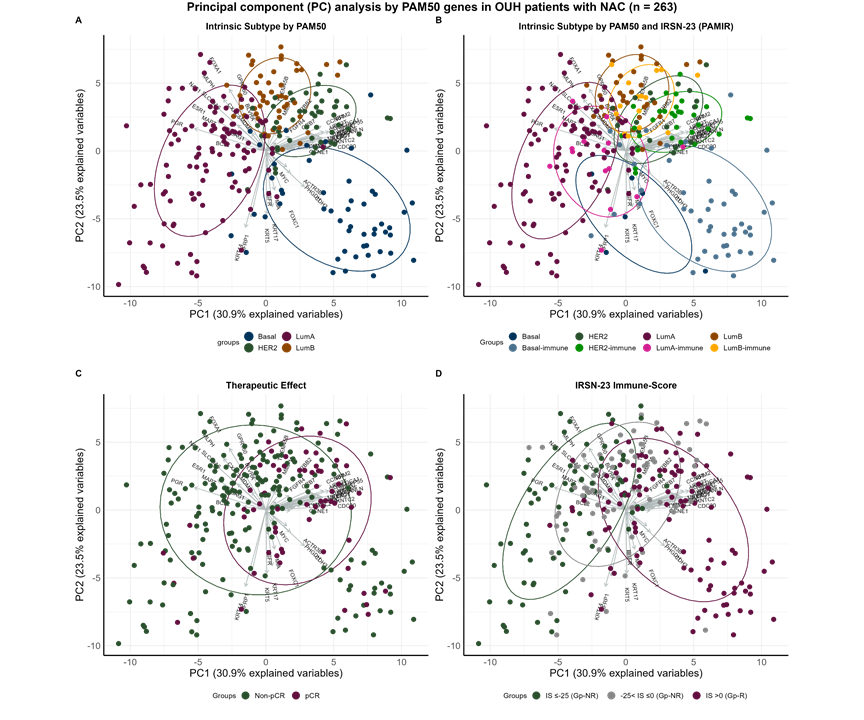


**Figure S12. Prediction of pCR by PAMIR in all validation datasets without anti-HER2 therapy using Affymetrix DNA microarray**

Bar graphs showing pCR rate (y-axis) and IRSN-23 (x-axis) in (A) Basal, (B) HER2, (C) LumA, and (D) LumB subtype by PAM50, respectively.


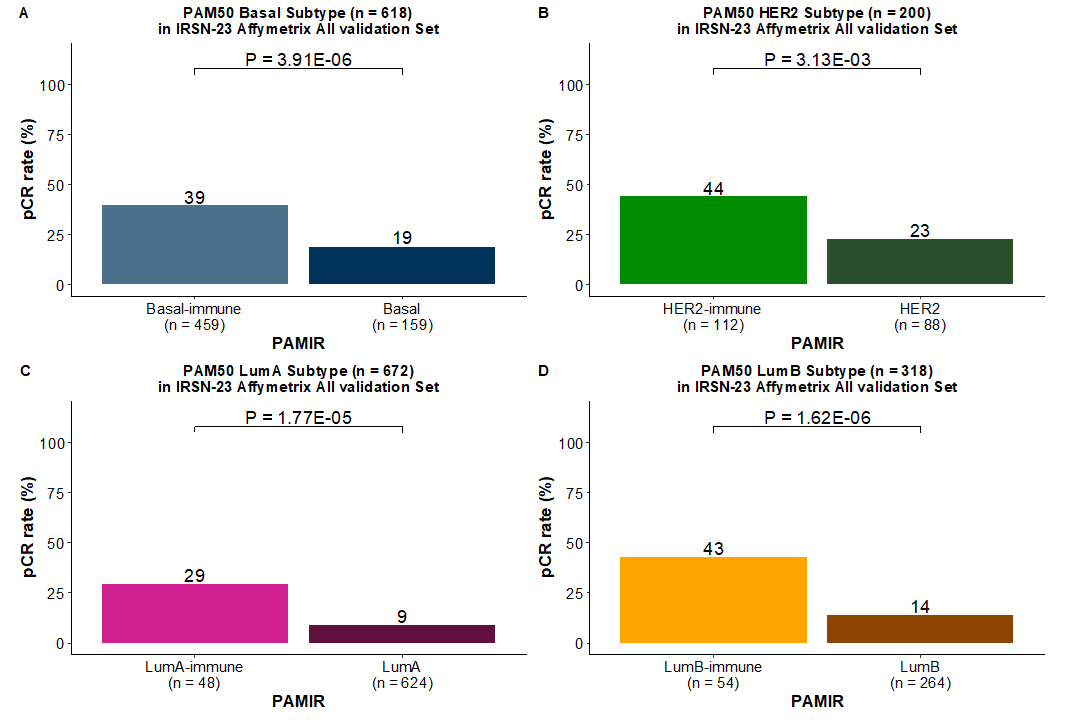


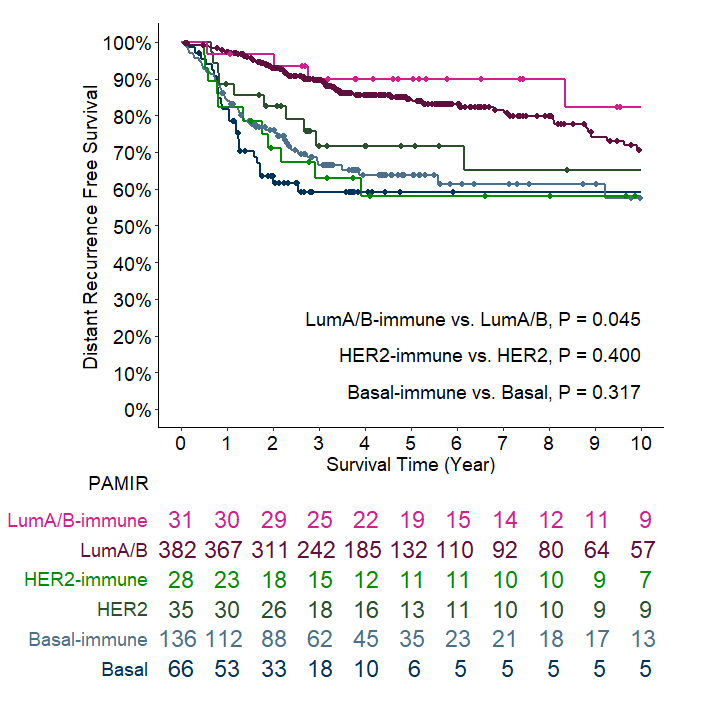
**Figure S13. Subtyping of PAMIR (PAM50 and IRSN-23) and disease-free survival of non-pCR cases after neoadjuvant chemotherapy**

**Figure S14. Correlation between microarray and RNA sequence in IRSN-23**


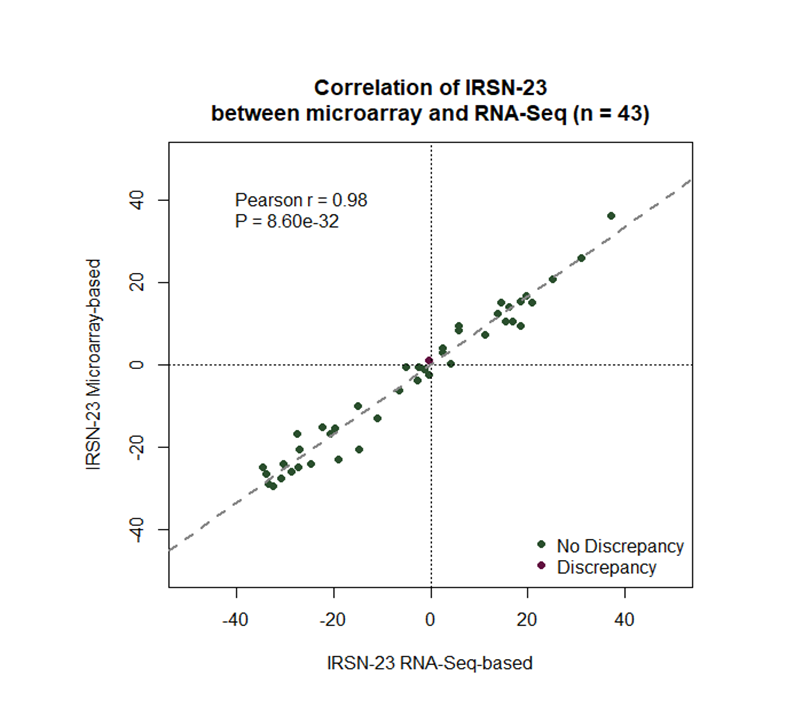
Purple dots indicate IRSN-23 results with different diagnostic results between DNA microarray and RNA-Seq, and the same results are indicated by green dots. The correlation coefficient was calculated using Pearson’s correlation coefficient.


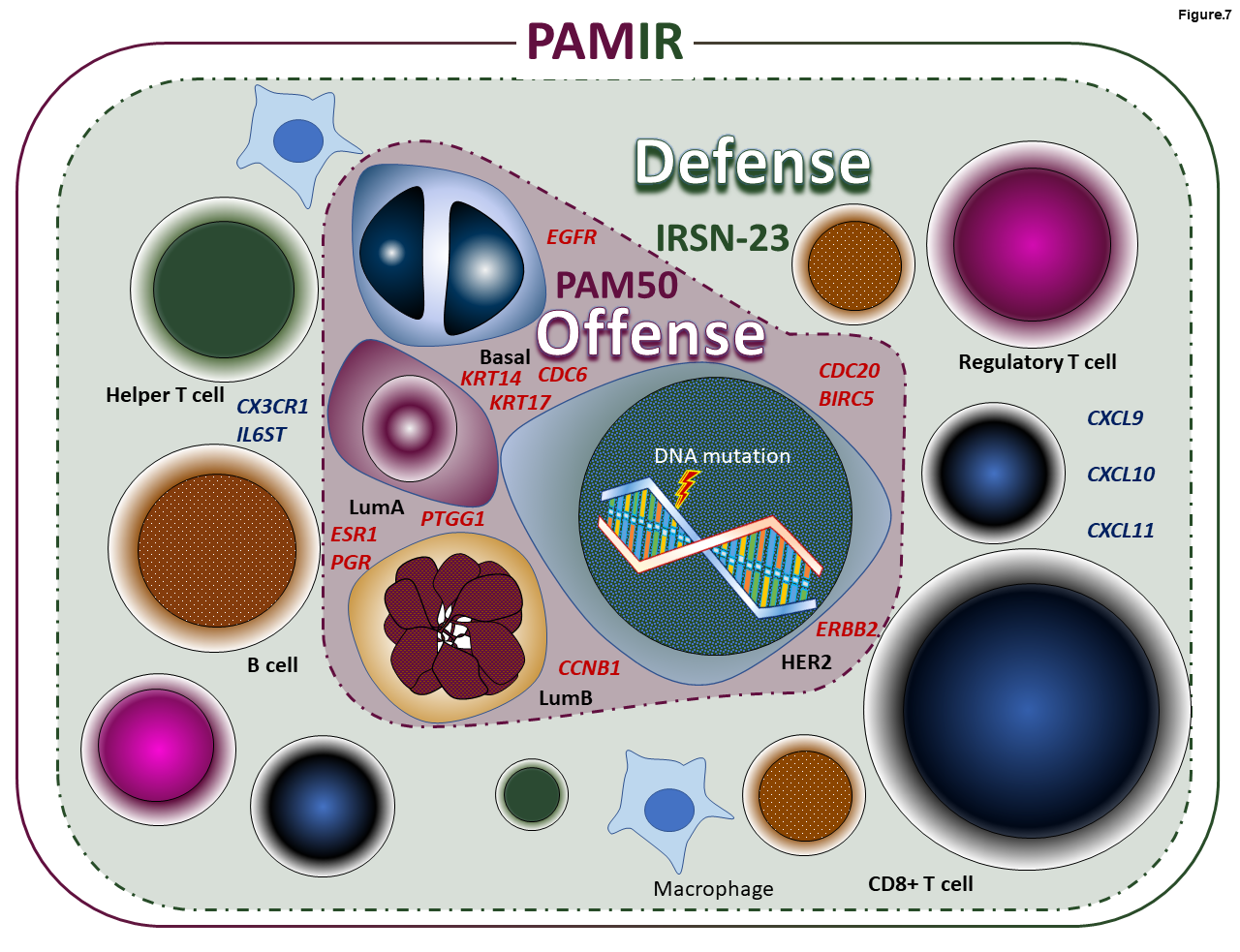
**Figure S15. Concept of new breast cancer classification as PAMIR.**
