# Supplementary material for: IRSN-23 gene diagnosis enhances breast cancer subtype classification and predicts response to neoadjuvant chemotherapy: new validation analyses
Source: Breast Cancer. 2025 Mar 24;32(3):566–81. doi: 10.1007/s12282-025-01687-6 (PMC11993443; doi:10.1007/s12282-025-01687-6)
Supplement: Supplementary file 2 — (DOCX 21 KB) [file 12282_2025_1687_MOESM2_ESM.docx]

## Table S1

## pCR Prediction Accuracy Table

| Dataset | DNA.microarray | Anti-HER2 Therapy | Number of Patients | Accuracy | Sensitivity | Specificity | PPV | NPV |
| --- | --- | --- | --- | --- | --- | --- | --- | --- |
| OUH Validation II | GPL570 | No | 112 | 72% | 92% | 70% | 29% | 99% |
| GSE25066 | GPL96 | No | 488 | 71% | 67% | 73% | 38% | 90% |
| GSE28844 | GPL570 | No | 32 | 69% | 100% | 64% | 29% | 100% |
| GSE42822 | GPL96 | No | 66 | 71% | 52% | 83% | 65% | 74% |
| GSE140494 | GPL570 | No | 91 | 66% | 61% | 68% | 39% | 84% |
| GSE4779 | GPL1352 | No | 102 | 66% | 67% | 65% | 54% | 76% |
| GSE21974 | GPL6480 | No | 32 | 78% | 88% | 75% | 54% | 95% |
| GSE34138 | GPL6884 | No | 177 | 67% | 79% | 65% | 30% | 94% |
| ***Pooled Analysis*** |  | ***No*** | ***1103*** | ***69%*** | ***67%*** | ***70%*** | ***40%*** | ***88%*** |
| OUH Validation II | GPL570 | Yes | 34 | 59% | 58% | 60% | 78% | 38% |
| GSE37946 | GPL96 | Yes | 50 | 54% | 33% | 78% | 64% | 50% |
| GSE42822 | GPL96 | Yes | 25 | 36% | 8% | 62% | 17% | 42% |
| GSE66399 | GPL570 | Yes | 88 | 64% | 56% | 67% | 43% | 77% |
| GSE130788 | GPL6480 | Yes | 110 | 59% | 55% | 62% | 52% | 65% |
| ***Pooled Analysis*** |  | ***Yes*** | ***304*** | ***59%*** | ***48 %*** | ***66%*** | ***49%*** | ***65%*** |

*Note:* GEO: Gene Expression Omnibus; GSE, Series accesion number in GEO; GPL, Platform accession number in GEO; GPL570, Affymetrix Human Genome U133 Plus 2.0 Array; GPL571, Affymetrix Human Genome U133A 2.0 Array; GPL96, Affymetrix Human Genome U133A Array; GPL6884, Illumina HumanWG-6 v3.0 expression beadchip; GPL1352, Affymetrix Human X3P; GPL6480, Agilent-014850 Whole Human Genome Microarray 4x44K G41
