# Supplementary material for: IRSN-23 gene diagnosis enhances breast cancer subtype classification and predicts response to neoadjuvant chemotherapy: new validation analyses
Source: Breast Cancer. 2025 Mar 24;32(3):566–81. doi: 10.1007/s12282-025-01687-6 (PMC11993443; doi:10.1007/s12282-025-01687-6)
Supplement: Supplementary file 3 — (DOCX 22 KB) [file 12282_2025_1687_MOESM3_ESM.docx]

## Table S2

## Principal component analysis with PAMIR (PAM50 + IRSN-23) gene expressions and Enrichment Analyses by DAVID Bioinformatics Resources

| Genes | KEGG PATHWAY, GO Term | *P* |
| --- | --- | --- |
| **PC1 29.8% explained** |  |  |
| CX3CR1, CXCL10, CXCL11, CXCL9, IL6ST | hsa04061:Viral protein interaction with cytokine and cytokine receptor | 7.00E-05 |
| CDC20, CCNB1, PTTG1, CDC6 | hsa04110:Cell cycle | 2.93E-03 |
| CDC20, CCNB1, PTTG1, PGR | hsa04114:Oocyte meiosis | 3.20E-03 |
| CX3CR1, CXCL10, CXCL11, CXCL9, IL6ST | hsa04060:Cytokine-cytokine receptor interaction | 4.14E-03 |
| CX3CR1, CXCL10, CXCL11, CXCL9 | hsa04062:Chemokine signaling pathway | 9.27E-03 |
| BIRC5, PGR, CDC6, ESR1 | hsa05207:Chemical carcinogenesis - receptor activation | 1.21E-02 |
| CXCL10, CXCL11, CXCL9 | hsa04620:Toll-like receptor signaling pathway | 2.36E-02 |
| **PC2 18.4% explained** |  |  |
| KRT17, KRT14, PGR, ESR1, EGFR | hsa04915:Estrogen signaling pathway | 2.31E-05 |
| ERBB2, PGR, ESR1, EGFR | hsa05224:Breast cancer | 8.37E-04 |
| ERBB2, ESR1, EGFR | hsa01522:Endocrine resistance | 7.24E-03 |
| ERBB2, ESR1, EGFR | hsa05205:Proteoglycans in cancer | 2.95E-02 |
| PGR, ESR1, EGFR | hsa05207:Chemical carcinogenesis - receptor activation | 3.13E-02 |
| ERBB2, EGFR | hsa05219:Bladder cancer | 5.36E-02 |
| ERBB2, EGFR | hsa05213:Endometrial cancer | 7.51E-02 |
| ERBB2, EGFR | hsa05230:Central carbon metabolism in cancer | 9.00E-02 |
| ERBB2, EGFR | hsa04520:Adherens junction | 9.12E-02 |
| ERBB2, EGFR | hsa05223:Non-small cell lung cancer | 9.25E-02 |
| ERBB2, EGFR | hsa05212:Pancreatic cancer | 9.74E-02 |

**PC3 10.2% explained**

CDC20, CCNB1, PTTG1, CDC6 hsa04110:Cell cycle 3.93E-03

| CDC20, CCNB1, PTTG1, PGR | hsa04114:Oocyte meiosis | 4.29E-03 |
| --- | --- | --- |
| CXCL10, CXCL11, CXCL9 | hsa04061:Viral protein interaction with cytokine and cytokine receptor | 2.66E-02 |
| CXCL10, CXCL11, CXCL9 | hsa04620:Toll-like receptor signaling pathway | 2.86E-02 |
| KRT17, KRT14, PGR | hsa04915:Estrogen signaling pathway | 4.79E-02 |
| CXCL10, CXCL11, CXCL9 | hsa04062:Chemokine signaling pathway | 8.56E-02 |
| **PC4 6.7% explained** |  |  |
| CXCL10, CXCL11, CXCL9, IL6ST | hsa04061:Viral protein interaction with cytokine and cytokine receptor | 5.80E-04 |
| KRT17, KRT14, PGR, ESR1 | hsa04915:Estrogen signaling pathway | 1.48E-03 |
| CXCL10, CXCL11, CXCL9, IL6ST | hsa04060:Cytokine-cytokine receptor interaction | 1.25E-02 |
| CXCL10, CXCL11, CXCL9 | hsa04620:Toll-like receptor signaling pathway | 1.31E-02 |
| ERBB2, PGR, ESR1 | hsa05224:Breast cancer | 2.52E-02 |
| CXCL10, CXCL11, CXCL9 | hsa04062:Chemokine signaling pathway | 4.12E-02 |
