# Supplementary material for: IRSN-23 gene diagnosis enhances breast cancer subtype classification and predicts response to neoadjuvant chemotherapy: new validation analyses
Source: Breast Cancer. 2025 Mar 24;32(3):566–81. doi: 10.1007/s12282-025-01687-6 (PMC11993443; doi:10.1007/s12282-025-01687-6)
Supplement: Supplementary file 4 — (DOCX 20 KB) [file 12282_2025_1687_MOESM4_ESM.docx]

# Table S3

## Principal component analysis with PAM50 gene expressions and Enrichment Analyses by DAVID Bioinformatics Resources

| Genes | KEGG PATHWAY, GO Term | *P* |
| --- | --- | --- |
| **PC1 30.8% explained** |  |  |
| CDC20, CCNB1, PTTG1, CDC6 | hsa04110:Cell cycle | 1.44E-03 |
| CDC20, CCNB1, PTTG1, PGR | hsa04114:Oocyte meiosis | 1.57E-03 |
| BIRC5, PGR, CDC6, ESR1 | hsa05207:Chemical carcinogenesis - receptor activation | 6.15E-03 |
| **PC2 23.5% explained** |  |  |
| KRT17, KRT14, PGR, ESR1, EGFR | hsa04915:Estrogen signaling pathway | 2.31E-05 |
| ERBB2, PGR, ESR1, EGFR | hsa05224:Breast cancer | 8.37E-04 |
| ERBB2, ESR1, EGFR | hsa01522:Endocrine resistance | 7.24E-03 |
| ERBB2, ESR1, EGFR | hsa05205:Proteoglycans in cancer | 2.95E-02 |
| PGR, ESR1, EGFR | hsa05207:Chemical carcinogenesis - receptor activation | 3.13E-02 |
| ERBB2, EGFR | hsa05219:Bladder cancer | 5.36E-02 |
| ERBB2, EGFR | hsa05213:Endometrial cancer | 7.51E-02 |
| ERBB2, EGFR | hsa05230:Central carbon metabolism in cancer | 9.00E-02 |
| ERBB2, EGFR | hsa04520:Adherens junction | 9.12E-02 |
| ERBB2, EGFR | hsa05223:Non-small cell lung cancer | 9.25E-02 |
| ERBB2, EGFR | hsa05212:Pancreatic cancer | 9.74E-02 |

**PC3 11.4% explained**

| KRT17, KRT14, PGR, | ESR1 | hsa04915:Estrogen signaling pathway | 6.96E-04 |
| --- | --- | --- | --- |
| **PC4 8.6% explained** |  |  |  |
| KRT17, KRT14  KRT17, KRT14 |  | hsa05150:Staphylococcus aureus infection  hsa04915:Estrogen signaling pathway | 4.60E-02  6.56E-02 |
